# Supplementary material for: Molecular and Functional Analyses of Characterized Sesquiterpene Synthases in Mushroom-Forming Fungi
Source: J Fungi (Basel). 2023 Oct 14;9(10):1017. doi: 10.3390/jof9101017 (PMC10608071; doi:10.3390/jof9101017)
Supplement: Supplementary file 1 [file jof-09-01017-s001.zip › jof-2583866-supplementary/File S1.pdf]

>LdSTS1
MSLSPSYFILPDLFSHSTAFHDATNPFWKRASAESRRWVNSYTVFVDRRRAFFFQGQSELL
SSHCYPYAGYEEFRTCCDLINLLFVIDELSDEQCYGDARRTGDIFLQAMRDPTWSDGSKLA
QMTADFRERLVTTVKPKTFRRFMEHCDAYVDSVVEEAGLRDRGEILELEEYVHLRRENSA
VRVCFGLISYILGIDLPDEVFSDPTFQKMYFSAVDMVCWANDLYSYNMELNRGLEGNNFIT
VLMKTQNMDIQAACNFVGGHYKQLMDDFLSAKASLRSFGSKVDVDVRRYIEACQHWPV
GNLVWSFETPRYFGARRDQIRRTRVVPLKPLEREPLSEDD
>LdSTS2
MSGLPQFYLPDLVAQWPWPRILNQHYEEAKPESDEWLRGFEALDVKSQRSFDRCNLALL
SSLGYPSLGKDCLRVACDLMVLFFIYDEYTDKLDGDGARDCADIVMRALRNPHKERSQG
ESKLGEITRQFWLRAIKVASESAQRRFLKSFAAFVYAIIDEASDRNDGRIRSITDYLELRRLT
VGAYASFFSLELGLDIPDEVMTHPAMESLLGLVTDSIVLTNDVNQDLYSYNNEQAAGHGG
HNILTVVMNEKGVDLDGALDWLAEYNGAILSRFQAQYRMLPSWGPDVDPIVTTFVERLG
HWIRGHDCWSFESERYFGTKGPEIQKHRVVTLLPRSTRPDVTPMMAQPVL
>LdSTS3
MHSSANSFYLPDLLVLSKPFKGSTNPHYKKAAAESRAWVNSYNIFTDRKRAFFIQGYNEL
LVSHTYPHAGYEEFRTICDFVNLLFVVDEVSDDQNGADARRTGEVYLNAMKYPGWTDGS
ALAKMTGEFRARLTRTSGPFSFRRFLKHCQDYIECVAREAEYRERGEVLDMESFKHLRRE
NSAIRLCFGLFEYSLGIDLPDEIFENPVFESLYWAAADMVCWANDVYSYNMEQAKGHTG
NNIVTVLMKAHGCDLQTASDIIGAHYAELMERYLADRERLPSFGVEIDADVQLYVRAMEN
WPIGNLEWSFETNRYFGPLHDEVKRTRLIVLTPRRDDSDTDSD
>LdSTS6
MSSADSSPTLSPSSTPPDSPLSSTRALSPTPTIHAPAFFVLPDLVSHCPFSPVYHDDGDAVAA
ESLDWILSYVQHFAQDKVAAMCGLQAGELTAYCYNNCSRDRLRVVSDFMNYLFHLDNVS
DGFLARDAAGLADWVMNAFEWPDSYRPVQGQQGGVEEISAAKLARDYWSRCIRDCAPA
VQQRFKSSMQMFFQAVHQQALFRANGVVPDLETYIDMRRDTSGCKPVFDLIEYSLDLELP
DVVVEDPVIVALNQGANDLVTWSNDIFSYNVEQSRGDTHNMICVYMIHDGLSLQQAVDR
VGNMCKRTIETFVENQARIPSWGDGIDEDVKLYVHGLREWIVGSLHWSFVTTRYFGDDG
EFVRTTRIVDLLTQEEDAKGEVVDLSC
>LdSTS7
MSELPQFHLPDLLAQWPWPRLSNQHYEETKPAFNEWVRSFEALDPKSQSAFDRCNFALLS
SIGHPLLNKDCLRVTCDLMALYFLYDEYTDKLDEDGVRTCADVVTDALRNPHKERPQGE
SKLGEIARQFWLRAIKVASEGAQRRFLKSFTTYAYSIIEETSDRNSGRVRSIADCLELRRRTS
AVYSTLFCVELGLDIPDEVMTHPAMVSLLALAIDPLMLTNDLYSYNIEQAAGHGGHNILTA
IMNEKGVDLDGALDWFAEYNGAILSKFQAQYRMLPSWGPDLDPIVTAYVERLCYWIRGH
DCWSFESERYFGKKGPEIQKHRVVTLLPRSTRPDVTPMMAQPVL
>LdSTS8
MSERYYIPKTLENWKWPRRINPHHNEVKAAAEAWIRGFRAFSPKAQEAYDRFLLASLGYP
WHDKARLRTAGDLANLIFVYDEFSDAGNEIQVQAMANVMVDALRNPHIPRPQGEWIGGE
ISRQFWELAIKTASPQAQKRFIKVFDAYAQAVVQEAADRGHKYVRSVHEYLEVRRDTIGG
RPAFSIIELGLNLPDEVVEHPAIEDMTAWAVDMLILDNDIASYNVEQARGDDGHNIVTIVM
HEHKTDIQGAMNWIHSYHKELEAKFMDRYENGIPTFGGPVDTELARYLDGIGNFVRAHE
QWNFETER
>LdSTS10

MRSHDPTNKLLIPIFPPAYPRRILAATRHIHTGDNTMPERYYIPKTLENWKWPRRINPYHNE
VQAAAVAWIRSFGVFSPKAQEAYDRFLFASLAYPLHDKARLRTGCDLVNLIFVYDEFSERG
NEGEGRMGWRGNRQTIYSRFWRLAIKTASPQAQKRFIKVYDAFAQALVQEAADRDHEHI
RGVQEYFELRRDTISARPAFALIELDMNLPDEAVSHPVIEEMTILAIDMIILDNDIASYNVEQ
ARGDDGHNIITIVIHKHKTDIHGAMNWVHNRHKELEAKFMDLYENKIPNFGEPVDTELLR
YVDGIGNLVRANDQWNFETERYFGKKALEIGRTRWVTLLPKERPEYIGPLLVDGSLL

>LdSTS11

MSEFRLPNLLAQWPWPRAINKHYEEIKVQFDEWVHAFGALDTKSQKLFDRCNFALLASL
GYPLLDKECLLVACELMALFFIYDEYTDKLDEAGARICADAVMDALRDPHKERPQDEPKP
GEIAKQFWLHAIKVASDSTQRRLIDSFGPYVYAVVEEAADRDQGRIRGITDYLDLRVRASA
AYCTLFPLEMGLDIPDEVMTHPAMKTFLDLIVYPLCLTNDLYSYNIEQAVPGHAAHNLLTV
VMNEKGVDLNGALDWYTEYNEAKLAKSLEQHRKLPSWGPEMDLAVTTFVEGVGYWM
RGHDCWSFEAERYFGAKGPEIQKHRMVTLIPKSTEITPMMALPSAC

>LdSTS12

MLELPQFCLPDLFAQWPWPRLSNQHYEETKPESDEWVRSFEALDARSQSAFDRCNFALLS
SIGHPLLDKDCLRVTCDLMALFFMYDEYTDNLDEDGVRTCADVVLDALRNPHKERPQGE
SKLGEIARQFWLRAIKVASEGAQRRFLKSFAEFAYSIIEETSDRNAGRVRSITDYLELRRRTS
AVYSTLFSVELGLDIPDEVMTHPAMVSLLALAVDPLMLTNDLYSYNVEQAAGHGGHNLLT
AIMNEKGVDLDGALDWFAEYNGLTRDVPEPVPTPGSDELLIDVHSAGLNFFDSQGKYQT
QPPRPFVLGAEFAGTVATAPPGSPYKPGDRVFGCCQGAYGERVVAKPVDVLPLPDTLSFDQ
GAGEWLLVLAAAGGVGIAAIQIGKVLGARVVACASPSKLNVARTVGGADFVVDYTKDG
WQKEVLKITGGRGVDLVYDPVGRIKDALKCTVWGGRALVVGFAGAST

>LdSTS14

MSNTQFSLPDPLARWPWPRKLNQHYAEVKPESDQWIHGFEALDPKSQRSFDLCNFSLLGS
LVYPLLDKDGVRVGCDLMVLFFIYDEFTDKVDGDGARVYAEMVMDAIRDPHKERPQGEP
KLGEIARQFWLRAMKISSPEAQRRFITTFAEYVYAVIDEASDRANGRVRGVEDYLKLTRLT
AGGYPSFLAAEAGLNIPDEVMAHPALQTILSLAAESLVLTNDMYSYNIEQASGHGGHNIVT
VIMNEKRVDLDGALNWLAEYHGQVLSNFQAQHRLLPSWSPEVDADVSTFVERLAYWIR
GIDCWSLETERYFGTKGPEIKEHRRITLLPKVKKPDVTPMMAQLNA

>Cop1

MSSLDATIHPVLNFEDKKIVLPDLVSHCNFKLRVSRHRKRITGETKRWLFKGDNLVGPARN
KYHGLKAGLLTAMTYPDAAYPQLRLCNDFLTYLFHIDNLSDDMDNRGTWSTANEVLNSL
YHPYTYHGQARVGRMTRDYWRRMILTASPGSQQRFIETFDFFFQSVTQQAIDRLTGEIPDL
ESYIALRRDTSGCKPCWALIEYANNLDLPDEVMDHPVVRSLGEAANDLVTWSNDIFSFNV
EQSKGDTHNMIPVVMHQEGLDLQSAVDFVGEMCKSAIDRFIEDQNYLPSWGPKIDRDLA
VYINGLADWIVGSLHWSFETERYFGKNGRQVKSSRVIDLLPRRSQ

>Cop2

MPSPAGALPKSFILPDLVNDCPFPLRVNPLCDEVGRLSEQWFLRHANYSPPRAVAFMALKA
GELTAACYPDADAFHLRVSDDFMNFLFNADDWLDDFDIEDTYGLANCTVRALRDPVNFI
TDKRAGLMTKSYFSRFLKTAGPRCTERFIQTLALYFESVVTQKQARNNGTLPDLESYITIR
RNNSGCKPCYALIEFCAGIDLPDEVINHPIIQSLEDASNDLIAWSNDIFSFNREQSRHDSFNM
VSIVMHQKGFALQEAVNFVGELCKKAMERFQADKRNLPSWGPEIDGEVAMYVDGLQNW
IVGSLNWSIDGTERYFGKDGPGIKKHRKVKLFPKRPLKTPAVRVLA

>Cop3

MSTPSSSLTTDESPASFILPDLVSHCPFPLRYHPKGDEVAKQTVHWLDSNCPDLTAKERKA
MYGLQAGELTGYCYPYTTPERLRVVADFLNYLFHLDNISDGMMTRETAVLADVVMNAL
WFPEDYRPTKGQAAEELNPGKLARDFWSRCIPDCGPGTQARFKETFGSFFEAVNIQARAR
DEGVIPDLESYIDVRRDTSGCKPCWVLIEYALGIDLPDFVVEHPVIAALNQGTNDLVTWSN
DIFSYNVEQSKGDTHNMIIILMEHHGHTLQSAVDYVGSLCQQTINTFCENKQQLPSWGPEI
DDMVAKYVQGLEDWIVGSLHWSFQTRRYFGDEGQEIKQHRLVKLLTVAPPPPPPPTPPPQ
SSDADTKKQKVKAQDGKGPVSDEEVWALVRAEQSKGSILESLFGFLTTSLSRIFFGYFFAY
SH
>Cop4
MRPTARQFTLPDLFSICPLQDATNPWYKQAAAESRAWINSYNIFTDRKRAFFIQGSNELLC
SHVYAYAGYEQFRTCCDFVNLLFVVDEISDDQNGQDARATGRIFVNAMRDAHWDDGSIL
AKITHEFRERFVRLAGPKTVRRFADLCESYTDCVAREAELRERNQVLGLNDFIALRRQNS
AVLLCYSLVEYILGIDLDDEVYEDPTFAKAYWAACDFVCWANDVYSYDMEQAKGHTGN
NVVTVLMKEKDLSLQEASDYIGRECEKQMRDYLEAKSQLLQSTDLPQEAVRYIEALGYW
MVGNLVWSFESQRYFGAQHERVKATHVVHLRPSSVLEASCDSDSDSDC
>Cop5
MVGSYTGKVIHVPALLESWPWPAAINPLYEQVQEESTSWFRKFDLYRDRKKQAIHDHLDT
AKFGASVCPKADYALLRLATDYLHLGFWIDYFFDTSPSDVIRQLTESIAHLLESGDPRLDSS
SPQSHIACMEILRDFRKRIETFNPSQEDLRRFVKEYRGFLEAELTQAIDHENKVIRDIESYLS
IRRSTIAIRPGIALLGLALGIPQEILDDPYTDTLTNACLDMVIIQNDAYSWNVEQVRKADGH
NIITVLMKQRDIDVQEAYEHAAQLHRETQEHFLELHAKRPDWGNEGSIQAFFDGLGEFVR
GVDEWSSMCLGEHALSVGAGFLK
>Cop6
MPAALPYNVSRDNKWDIKKIIQDFFKRCDVPYQVIPYDTELWNACLKRAKEKGYPVEPDS
PMSLYRSFKVGVVITRTSYGHIQDYEILIWVATFTAFVTYADDAFQEDIQHLHSFARTFLQN
EKHEHPVLEAFAQFLRESSIRFSHFVANTVVSSALRFMMSIALEFEGQNVSVSTEAREYPG
YIRILSGLSDIYALFAFPMDLPRSTYIQAFPEQIDYINGTNDLLSFYKEELDCETVNFISAAAT
SQQVSKLEVLRNAAEKAAYSYDVVVNVLKPYPEALAAWKSFARGFCYFHTSSPRYRLGE
MFHDFEHDLVCKCASCTEI
>Omp1
MKYTSFALPDLASSCDYNLRFNKYHRSVSPETKKWFFRLSPASQADLTTYDAQRFTLLAA
VCYPDAGYPQLRVCSDFLAYLFYLDNLTDDMDDKSTRSVADLVLNSLNEPETFQTQYRIG
KMTSDYFKRIIQTSNDGTKKRFIDTMDSFFKSVDDQARDRLAGHIPDLESYIALRRETSGC
KTCFSLIEYANNLHIPDEVISHPHIEQMETAANDVVSFANDIYSFNIEQSKGDTHNMIPVLM
HANPDMDFLEAVSFVRDLTIKAMDRFNELRATLPSWGLDIDKDMKVYVNGLENWMVGI
LFWSFETERYFGKSVRSVKATKTVNLLPSRA
>Omp2
MASTAPSKFILPDLVSHCSFDLHHNRHRKQITTETKKWLFKGDNLTGRKRDQYHGLKCGL
LSAMCYPNAAYPQLRVCNDFLTYLFHLDNLSDDMDNRGTTTTADVVLNSLYHPGYFQSA
RVGKMTRDYWKRLISTASPGTQQRFIETFDFFFQSVTEQAHDRQAGVIPDLESYIALRRDT
SGCKTSFVLIEYANNLDIPDGVMDHPLIRSLGEAANDLVTWSNDIFSYNVEQAKGDTHNM
IPVIMNEHGLDLQSAVDYVGRLCQQSIDRFISDRAQLPSWGPEIDRQVAIYVDGLTDWIVG
SLHWSFESERYFGKSGRQIKKSRVINLLPRRA
>Omp3

MAIENTIASAPASTPAKQLDTPDHFILPDLVSHCTFPLVYHSNGDAVAAQSVKWLDTNCPD
LNDKRRKALYGLQAGELTAYCYNTAPDQRLRVVSDFMNYLFHLDNISDGMMTKDTDALS
DAVMNALWFTEWYRPTKKSDYVQPDEELNAGKLARDFWHRCIQDAGPGCQARFKETLE
LFFEAVNIQAKARDAGVIPDLESYIDVRRDTSGCKPCWALIEYGLGIDLPDYVAEDPIIKSL
NQSTNDLVTWSNDIFSYNVEQSRGDTHNMIVILMLYHGHNLQSAIDYVGDLCRQTIDDFK
ENRKKIPSWGPEVDDIVKQYVQGLQDWIVGSLHWSFMTTRYFGKQGQEVKKNRYVKLL
PVGEEANKW
>Omp4
MYIFCSQVVSGLNKFTFKMSSAPTRFLLPDLLSACPLKGSVNPYYKEAGAESSAWINSYDI
FTDRKRAFFVQGCNELLVAHTYPYAGYEEFRTCCDFINVLFVLDEVSDEQSGSDARFTGEV
FLNALRNPENDDTSKLSKISKEFRARYFKRAGPRTAERFLQHCQDYIDCVTREAELRERGE
VLDLPSFTALRRENSAIRICFCLFEYALGFDLPQEVFDDPTFMEMYWAAADLVCWANDVY
SYNKEQAQGHGGNNIVTVLMKAKDLDLQAACDYIGVYCEELMGRYLSAKARLPSWGPE
VDAAVAQYVEASGHWVRGNLDWSFETQRYFGAQHAEIKETRLVTLTPAIPEDFSDTGSES
E
>Omp5a
MSPDPTRIVLPDFLAACPFESSTKNPHLKAAGAESSAWVNSHVVFNDRKRAAFMQDIYEL
LVAYAFPWADYEDFRTMCDFINLLFVLDELSDDQNGKDAGYTGKLFMDAMRNIDNGDTS
ELTELCREFKARYSKRVSPQVNERFLQHLQSYTDCVAQEADLRERGEILDLESYVALRREN
SAIRPCFDLVEYIIDFDIPQEVIDHPVFSEMYWASVDLVCWSNDVYSYNVEQAKGHGGSN
VVTVLMKEKNLDLQAACDYVGVYYEELMDRYLSAKARLPSWGPEIDAAVGKYILAEAQ
FVRGNLDWSFDSPRYFGPQHDQVKKTGIVTLTPAPKKFGSDSGSESE
>Omp5b
MSPAPSRIVLPDFFASCPFESSTINPHFKAAGAESSAWVNSHVVFNDRKRAALMQNSYELL
VAYAFPWASYEDFRTLCDFINLLFVFDEVSDDQNGKDAGYTSKIFMDAMRNIDNGDHSEL
TELCKEFKARFSRRLSPQVNERFLQHLQSYTDCVAQEADLRERGEILDLESYVILRRENSA
VRPCFDLVEYIMDFDIPQEVLDHPVFSEMYWASVDLVCWSNDVYSYNVEQAKGHRGSNV
VTVLMNEKNLDLQAACDYVGVYYQELMDRYLSAKARLPSWGPEIDAAVGKYVLAEAQF
VRGNLDWSFDTPRYFGPQRDQIKKSRIVTLTPAPKKFGSDSGSESE
>Omp6
MIAKNSEIDRFYIPDTLANWPWPRHLNPAYPEAKKASAAWLRSFNAFNERSQKAFDLCDF
NLLASLAFPLADLYCLRSGCDLMNCFFIFDEYSDVADPQTVRQQADIIMDAIRNPHVPRPR
GEFIGGEAHRQFWERAMQGATPTAQRRFIDTYQQYTDAVVQQATDRADNHIRDVEGYFT
VRRDTIGAKPSFTLLEFTMDIPDEVMGHPVIKDLSLWCIDMLIIGNDLCSYNVEQAHGDDL
HNLVTIVMNQYNLDLPGAMEWIGKFHDDIADKFLDTFAKLPSWGPEIDPQIRRYVDGLGN
WVRGNDSWSFESWRYFRGKGPEIEKTRWVDLMPTEEATITPKYESDSNAAQPAQST
>Omp7
MPETFYLPDCLANWKWKRALNPNYPEVKAASSEWLRSFKAFPPKAQEAYDRCDFNLLAS
LAYPLADKDGLRTGCDLMNMFFVFDEYSDVAHESEVQVQADIIMDALRNPHKPRPVGEW
VGGEVTRQFWELAIKTASPQSQKRFIETFDTYTKSVVQQAADRTQHYVRTVDEYLEVRRD
TIGAKPSFAILELTMDIPDEVIHHPTIERLAILAIDMILLGNDTASYNYEQARGDDNHNMVTI
VMHQYKTDIQGALSWIEKYHKELEEEFMQLYNSLPKWGGQIDVDIARYVDGLGNWVRA
SDQWGFESERYFGTKAPEIQKTRWVTLMPKKRAEGVGPEIVDISEL
>Omp8

MSQILHLLWSKFSTSLPSTVTIGSDPQTLQLVHSPAPNVNANALEIRRIVNNFLTRCSIRLEG
TPLDVDFYNECKKTLLSHYIGIHDSDKVSESWFRRYLSVGVIITTNAYGHIDDKPTRVYICL
YTALLTSFDDVFEANVEQFGGFNERFMKGEPQEDLFLDALARILLDAPRYYGRLATNIIVT
ATLDFFTGLFLELQARDMTFNEDLHNFAVFCRNLTGIAHAYAVFMFPRDVPFTVYYVRSLPE
LKTDINYVNDIMSFYKEDRAEETDNLASILRQVHPSMTKHQVLQKIVDDAVEADVRARKI
LADYQPALDAYEHFRKGYAMFHVSSGRYRLDELFSYIRFE
>Omp9
MSQILHLLWSKFSTSLPSTVTIGSDPQTLQLVHSPAPNVNANALEIYKIVDNFLSRCGIRLES
TPLDVEFYNECKKTLLSHYIGIHDSDKVSESWFKRYLSVGVIITTNAYGHLDNKLTKIYIAL
YTALATCFDDVFEKNVDHMSGFNERFMKALPQGDVFLDAFAKVLLDAPRYFGRLASNIIV
TSTLDFITSMSVDVLTKGMKFNQNLHNFAMACRNMSGIAYTYAPFIFPKEVPFAIYAQCLP
DMRIYINHVNDVLSFYKEDKAGETENLASILGQVHPSMTKYQIVQGLADDAAEADLRVR
TVLSQYQPALDAYNCFRQGYVSFHASSGRYRLDELFSFVEPEPIV
>Omp10
MRNFLSQCQIPLQRGVPLDPTFHQECANVLIEDYLKPSAAVTLENLPSLMSSFNPFLTLGV
RMASTGYAHLTHTPTRVYVALFTALLVCLDDIFPENVELMCGFNERFIKNETQGEPILDAVA
GLLRSTSKYFSMLSSNLIVTSALNYVTSLSLDQGLHSIKATEHSRNFARLCRNMSGIPEAFA
AFVFPPEVPFTAYIQCFPDLYTYANYVNDVLSFYKEDIAGETENLVSILAQTQPNSSRYQVL
QQLADEAAAANANIRDILSDQKSILDAYDAFRVGFVQFHIDSPRYRLAELFPCIDG
>ShSTS1
MAHPTTNPSHRLEQMESVREHIPRLQHFLGEIGYRHTTPPAPTLDFLHAHHHWIHHVLGP
MTSWTVAKLNALEDSSSTIFERAYPLSDAEMKFVLAKLTAIAIFLDDSLEDEETYDDIGNFA
HRVYLGEAQPTGVLTLYHQGIQELSKMHEGDAVFRGLAVAPWITFIDACMLEKRLLTFDSK
LRVSPRDLGYQRLRNSTDFTSLRAPKATPSEVEVSFPIFLRHKSGIGEAYAAAIFKSSRYQEL
PLSRFVKSMPDMIYYIELVNDLMSFYKEQLAGETANLIHLQHQSWKGGQGTGPYGSWTL
LDTFSRLCDETRDAAFRVDELLRLDECEKIANGELRGEEVGLSPMDVTMAAQWREFRDG
YVSWHLECQRYKLDFIKLSTFE
>ShSTS11
MAAPESSAWVSSYNLFSDRKRTDFITGSNELLVSHTYPHADYDAFRTCCDFVNLLFVIDEI
SDDQSGKAARRTGEVYLNAMRDPEWTDGSDLAKMTQQFRARFLRSVGPQSFRRFLRHSE
DYIDCVAKEAEYRERGQVLDMDSFKSLRRENSAIRLCFGLFEFTLGIDLPDSVFEDETFMK
MYWASADMVCWANDVYSYNVEQAKGHSGNNIVTVLMAARDIDMQAASDYVGEYYAE
LMEEYMTAKAELASKSFGSRDLDEDVWKYVNAMENWPIGNLEWSFKTNRYFGTLHDEV
KRTRLVVIKPRKVVV
>ShSTS18
PRHINPHYQEVKKASAAWAESFGAFNPKAQHAYNACDFKRLRTGCDLMNMFFVFDEYSD
VSSPKDVIQQAAIIMDALRNPYAPRPDDEWVGGEVTRQFWKRAIKTATAGAQRRFIDAFE
SYTQSVVQQAKDRHHGFIRDVDSYLEMRRETIGAKPSFVVLQMDMTLPDEVLAHPVIQQ
LSALSTDMICLGNRRLIQILWTVQDICSYNVEQARGDDLHNIITIAMNQFDIDIAGAMDWV
VKYHAKLERKFLYLYNNGLPSWGKELDPQVERYVCGL
>ShSTS15
MVRSPVSDKFCIPDTLASWPYPRILNPHYAEEKAASAAWTKGFGAFGPKAQDAFDRCDFK
RCRSGCDLMNLFFVIDEHSDTHGEETVRKMKDVVMDAIRNPHKPRPNDEWIGGEIARQF
WERAMCYASEISQRRFIDTFDEYLESVVDQAADRDSARIRDIESYINIRRNTIGAKPSFVIM

EQGMDIPDNVFENEVFQRLRMATIDMLCLGNDIVSYNIEQARGDDSHNIVRIVMNELDTD
VPRAMDWVAQRHTQLEREFFTALSELPTWGEPIDGWVKEYVYGLGNWVRANDQWSFES
QRYFGTKGMEIMKSRWLSVLPKVRPAEVGPQLVDQSLL
>ShSTS16
MAVATSVATPVPTPAYSAGRAPAKEKKIYLPDTLAEWPWPRAINPHYAEAKEESQAWAASF
NAFSPKAQHAFNRCDFNLLASLAYPLATKHGCRSGCDLMNLFFVIDEYSDIAPVEEVRQQ
KDIVMDALRNPHKPRPEGEWVGGEVARQFWALTITNASAQSQKHFIETFDEYLDSVVQQ
AEDRSESRIRDIQSYIDVRRNTIGAKPSFALLELDMDLPDEVLAHPTIQSLSLATIDMLCLGN
DIVSYNLEQARGDASHNIITIVMNELNLDVNGAMRWVGDFHKQLEKQFFEAFNNLPKWG
NAELDAQIAVYCDGLGNWVRANDQWSFESERYFGARGLEIMETKTLAMMPIQRTEALGP
QLVDDSIL
>ShSTS3
MSTAKPEIPPMESFPPINVYPREAEIIKYCNNYVAEHFPFNNDAEVKHFNGMEIPAYACRVV
SFARDHEKMRKVSILIVFYFIFDDWVDKNGMKLDKSTVMALLPPPSEVPIKPPQAGKMTL
SDISAELYGAVRDDMPKADYDRMVNDMMEYLRVQRMAPGYNTLQELLDLRSGEVGVY
VLFRYIIYAMELSVSGKELDDPLVKRAQVLGSEAGVLRNEASSYVKEVNEGSGAHNVITK
LQEWSGCTEKEAMKQVLDAIEKRQEELREMCLKVTEAPHLSEDCKTFVKTIPYIVAGNT
WWHHHSTRYAEGREVTVP
>ShSTS4
MSETKVGKVAPFPPLPGAPYPPVRNHPRWKGAPAADFDRMVWGARIAGIFFLADDYIDSG
KMLDRIPGFKAVCTFLPLHKEDQAEICHDIVFRAIKATSHPRTFDQLTKCTHEWWDSNIHE
PFQNLDQYLAVRRVNIAMVTYFRYTLDINLTDEQVNHPLMREAEGIVSDHVGLTNDFFSY
LKEKMTNSDDTNIIRILMDHEHLSYEEAKTVIEKKIRQKEQDFIGAGMAVLNDPELGKDRE
IYRWIANLQYCMGGNLAWSQEVSENHYGIHVLDIT
>ShSTS5
MELSSLRPFPASFVLPNLANITRQAFNLKLNPHSQSANSAMKSWFKSFHVYDEQKSREFLE
AGKFDLYAALSFPDADLQHLETCLAFFFWAFSTDDLSDEGDLQSKPEEVQVGVDISTSALS
SHAPTSLDFPYAAMLQSLFNRIKTTATKGASERFIQAFKDWSSSQVMQSRNRSKLLLPSVE
DFILMRRNTIGAALVEAMIEYSLDLDLPDYVFRDPVVIAMSEATTDIMTWPNDLCSFNKEQ
ADGDYQNLVCCLMAQYDLGLQDAVDRLVGMISTRVRDYITLKEQLPLFGAEVDTMLRKY
HAALEHYVQGTIVWYYSSPRYFHGEQIIEKESTRIILFSKASSKDCS
>ShSTS7
MPHSTVHSHTLISDDSVLLFPDLISYCAYPLRVNPYGRSVADDSERWLLNGAHLSDKKRK
AFLRLRAGDLASMCYPDASAKSLRVVADYMNYLFKLDDWTDEFEAEDVDGMRDCVLA
ALRDPLHYETDKAVGKLAKSFFGRFVQHGGPLRTKRFIDTMVLFFRAVRQQALDRTYDDI
PDLESYIALRRDTSGCKPCFALIEFAGGYDLPDAVVEHPSIQILQDATNDLVTWSNDIFSYN
VEQSRGDTHNMVVVLMHEQGLAVQEAIDAVADLCERSIDTFEQTRRSLPSWGPIVDSNVE
SYIDGLQNWIIGSLHWSFLTERYFGKDRRDVKRKLFVKLLAKRC
>ShSTS8
MPAIIRQFILPDLFALSSAFPDATNPHWKRACTESRDWVNSYRVFSDEERRAFFTQGQSELL
CSHAYPYAGYEQFRTCCDFINLLFVLDEISDEQTADGAWATGRIFLQVLQDPEWDDGSKVA
QMTRDLRARVVSTGVKPHTFRRLVHMCRDYIASVVEEAGLRERGEVLDIESYIELRRNNS
AVLTCFALIPYILGIDLPDEVVNDPNFSALNLAAVDMVCWANDIYSYDMEQAKGLEGNNI
MTVLTEMHGLTMQDASDYVGEQYKALMDLFLDNQAALRSFGPSVDADVRRYVDAVRH

WPRGNLSWSFETPRYFREKREEIENTRVVILRPRPEPKMKSQN
>ShSTS10
MGATSTTQIPHSTSLESYILPDLLRLSSPFKASMNPHWATTASESSAWFSSYSIFSDQELTEF
AVCKVELLVAYAYPHANYETFRTCCDFMNLIFAIDEISDMQNGEDAGETGDVFLNALRDA
EWTDGSALAEMTRDFRARFLRSAGPQSFRRFLKVSEDYIDCVTKEAGYRERSQILDMESF
KHLRRDNSGVPLMLGLLEYTLGIDLPDVVFEDTALSRIYWAAVDMVWWANDVYSYKVE
QAKGLAGNNILSVLMAAKNIDLQDASDYVGDVYAGLMKEYTEAKAELVSKSFGSKELD
AAVKKYVDKMENWPIGNLEWSFASMRYFGSQSGEIKRTRTVMLRPQDVLA
>ShSTS12
MQTPPSCESYILPDLLHISQPFKASTNPHWLKAAPESSAWVSSYRIFTDRKRMEFILGSNEL
LASHTYPQANYDTFRTCCDFINLLFVIDEISDDQSGEGARLTGKVFLNAMRDPECTDGSIL
AKMTRDFRARLLQSIGAQSFRRFLKILEDYIESVTKEAEYREHGYVLDMEQFKILRRQNSA
VRCAFGMVEHTLGIDLPDVVFEDATFMKVYWAAIDMVWCANVNMFPPPGYLIQDVYSY
SMELAKGDNHSANNIMSVFMAANGVSLQAASDYVGRHYARLMEEYLSARAELASKSFG
SQELDSDVLKYIDAMENWPIGNIEWSFKTSRYFGPQNDEVKQTLVVKLKPQEVPA
>ShSTS13
MPDLFTAWPWKTTRNPLYLEAKADSDGWLATFGDFFQKYKDCILDCDGKRLRCAGDVN
TLLFVVDDILDYEDDATVKERMRILMDAMENPFKPRPDGEWIVGEMTRQFWERTIKVSN
TLSQERFMEGVRDCMAGMVRESLARKASRQHDSIHGFLKTRRDSAGCNLVFALSELEVA
VPAEAMKHSQIRELIAIIQDISCIANDTFSYRREQSRGRTEDNIITVVMNNLGTDIPGAIDWV
ENHHKELMKEFIEKHDKVPKWGEPTDSKVKNYIESLAGWVWANARWSFECRRYMGDEG
MEIMEKSRWVRAIPKERI
>ShSTS17
MSQTYTIPDTLANWPWKRKINQHYEEVKMESASWARSFHAFSPQAQDAFDRCDFKRLRT
GCDLMNLFFVIDEHSDLSSVADAETQAQIIMNALMNPEKPRPHGEWVGGEVARQYWELA
IKTATPKSQRRFVAAFDDYMNAVVQQAKDRTHSTIRDIDSYMEVRRKTIGAWPSFALLELD
MDLPEDFMDHPVMHELHVLSISMICLGNVSDIAPLPSDIVSWNLEQSRGDDTHNIVRIVM
NQLDTDINGAMAWVEMYHKELEVKFMDIFTKSQEWNKSMNKDISRYVEGLGNWVRAN
DQWSFESKRYFGDRGLEIMSKRTVSMMPKRNDVLNGTQLDIGPVIVDGSIL
>HS-HMGS
MSETKVGKVAPFPPLPGAPYPPVRNHPRWKELYRLHDEWMMKYWPFSSEKKRARIPFMN
LAGFSTWCAPAADFDRMVWGARIAGIFFLADDYIDSGKMLDRIPGFKAAATGTGPLHKE
DQAEICHDIVFRAIKATSHPRTFDQLTKCTHEWWDSNIHEPFQNLDQYLAVRRVNIAMYFA
NAYFRYTLDINLTDEQVNHPLMREAEGIVSDHVGLTNDFFSYLKEKMTNSDDTNIIRILMD
HEHLSYEEAKTVIEKKIRQKEQDFIGAGMAVLNDPELGKDREIYRWIANLQYCMGGNLA
WSQESGRYNVGVIDGISFPSLSYAAEPTPEDEVVDDTEESRLRELIFNVKDIPPPDFTIDDDA
IFMTNPHSHLQDNVVPLPRPENVGIIGLEVYFPKRCISIDALEDFDGVAKGKYTIGLGQQYL
AFTDDREDINSFALSAVSSLLEKYNIDPRSIGRLDVGTETLIDKSKSVKTTLMDLFAASGNH
DVEGIDSKNACYGSTAAVLNAVNWIESSSWDGRYAIVFAGDIAIYAEGPARPVGGAGAVAL
LIGPDAPLVLEPTHGSYMANTYDFYKPRMESEYPVVDGPSSVTTYITALDESFKAYQRKV
QEGSSRDVPPPYANGANGKASATKSVKLSDFDYSVFHSPYGKLVQKAYGRLTYHDFVAHP
TAPVYRDLPTDILSKDASATLTDKSVEKTFAAASASMYKQVVTPSLLISNRCGNMYTGSLY
GGLASLLTSIPSYELFDKRISMFAYGSGCASTFFAIKVRGDTSHIKAKLDLERRLAEMDVRP
CEDYVVALKLREETHNAPSYIPNDSDAGLWPGSYRLEAVDGKYRRSYTVTH

>CpSTS1
MPAAIPKFYTLPDTLRNWPWKREISPYYRQCQAESVAWLESFRPFSPKAQVAFNKCDFSLV
SALCFPKGSPYNLRSVCDLMHTFFTLDEYTDYLDLEGVKTLCEATMDAIRNPDKPRPEGE
HFIGEVARQFWARARVNATPACEERFVKSWRTYLNSVIQQAERRDAKYICTMEEYLHARR
DNIGSDPSFALLEITLEVDLPHEVMEHPTIVALARDTTDMIVLANDMCSYKKEIIADDANY
NAVTVVMHNHNTNVDGGIQWISDYHDTIVDHFLRLREDVRLKQNGFPSWGARIDREVEA
YVEGLGLWIRGHDEWNFGSGRYFGDEGLEVQKSRIVECTVSADPFTPFLIQQEEEVDENA
A
>CpSTS2
MMQFYLPALVEQCPIEGGTNPHYEQGAAESRAWINGYNVFTDRKRAFFILGSNELLCSHV
YYYAGSEEFRTSCDFVNVLFIFDELSDEQTGKDALETGQIFLNAMKDERWDDRSKFSSMT
KEFRKRFLRRSGPRGTARFLKHWETYCAAVIREAELREMDEVLDLEDFINLRRENSAVRL
CYGLIEYCYGIDLPEAVYEDSTFMDIYWAAVDLVCWTNDVYSYDMEQSKGIAGNNIVTVL
MRNRNMSLQQASNYIGQHCETLMDRFVSSQVRLPSWGPVVDREVRLYIQGLGAWIKGNL
DWSFETQRYFGPMHEEVKSTRLVTLRPRERIEECDSDSDSDFE
>CpSTS3
MVATTASTQPDHFVLPDLVSHCSFPLVYHTDGDRIAAQSVNWLDSNCPDLNAKQRVALRG
LQAGELTAFCYNTCTPERLRVVSDFMNYLFHLDNISDGMMTRETDVLADVVMNALWFSG
KYMPTKEQSADELNPGKLARDFWARCIPDAGPGCQARFKETLELFFEAVNIQARARDDD
VVPDLESYIDVRRDTSGCKPCWALIEYALDIDLPDFVVEHPIIEALNQSTNDLVTWSNDIFS
YNVEQSRGDTHNMIVILMKYHGHTLQSAVDYVGDLCQKTIDDFQANRQKLPSWGAEVD
EMVQRYVVGLQDWIVGSLHWSFQTHRYFGADGANVKKNRIVKLLPLKA
>CpSTS4
MSESNQHLRIPHTLTAWPWPRTLNPYYQTVKAESSAWLESFKAFDPKAQAGFNSCDFNLL
ASLAYPLASKEHLRTGCDLMNLFFVIDEYTDIEDEVHAAMVASVTMDALRNPFKPRPRGE
IIIGEIARQFWARTVPTITEASHRRFIETFDTYLQSVVVQARDRSKQHIRSIQDYLHMRRDNI
GAKPSFAILELSLDLPDYVMSHPSIQTATVTAIDMLIIGNDLCSFRNEHARGDDTHNILTIAR
HEFGQGLGHAVNWIESYNKSLRRSFLSAIERVPSWGEEIDTQVAEYLYGLANWVRANDC
WSFESHRYFGKHGREIQVHRVVDLACGHPECVQDTDNEVEKPATKGLLYEVEKKTYVGP
LQVNTKDPIAFRSDFPLSARPKDITSTPTRLTCVALLCTLSTFIGWLVKL
>CpSTS5
MSTIATGVALKSTRRPTKFILPDLVSHCTFKLRCSRFRQKVTTETKQWLFKGGNLGAKDRV
KFHGLKAGLLTAMTYPGAAYPQLRVCNDFLTYLFHLDNLSDDMDNRGTHTVGNVVMNS
LYHPHTYNEPERVGKMARDYFKRVIVTSSTGAQQRFRETMDFFFQSITEQALDRKNGVIP
DLESYITLRRDTSGCKPCWALIEYAYNLHIPDEVMEHPLILSLGEAANDLVTWSNDIFSYNV
EQSKGDTHNMIPVVMNQEGLDLQSAVDFVGQMCKQSIDRFVEDASNLPSWGPEIDRDVA
VYVNGLADWIVGSLHWSFESERYFGKTGLEVKATRVVELLPQRA
>CpSTS6
MLTTPYFTIPDTLRNWPWKRVLNPHQKVCEEEAADWMRSCGAFTPKSQNAFDRCSFGLL
GSLAYPRLGRDGLRIACDLMNMFFVIDEYSDVASGREARLQADIVMDALYNPLVPRPVGE
WIGGEVTRQFWANAIKTATPSSQRRFVRNFQRYVDAVVQQAQDREAHCIRDVKSYFILRR
QTIGAIPSLDLLTLEMDLEDEVLDHPIIAKLLELCVDMILIGNDLYSYNVEQARGDDTHNFV
RIVKDERKCNLNDALRWISDYHDRLADEFLNLMHNLPSFGSKLIDEQVKTYVDGLGNWV
RANECWSFESERYFGKKGKLYQKSRRVRLLPSSIALQGQNIDPQAAVEISDLPAAAGIIV

>CpSTS7
MTTSGFRIPNLVVAWPWLRAVNPHMDQTSQNSSSWLENFHAYPPATQENFNRCDFGLLAA
LTYPIASKEHLRTGTDFMNLLFFIDEITEIGDETFSTEVLYIAMDALRNPSKLRPKGEHVVG
EITRQFWERSIPHITATSHRRFVCALEAYLKSNVVQARDRSSQHIRRIEEYFSTRRDNIGTKP
AFVLLELGLDIPDCVMTHPSIQSAVTSLTDMLIIANDVFSFKVEHSRDDDANNVLKVVMCE
LRCNLARAIEWADERNQQLRTTFLSSIAEVPSWGGQVDIQVSEYLYGLANWVRANECWS
FESQRYFGTHGPTVQKDRWVSLDCGHPQCITGGTTTPRTSYRPYLVGMLGIATYALSQYG
LMSRATLLRR
>CpSTS8
MSNTFIVLPNLEETVYSAFPDHGLNPHYDAVCPHSRAWIKSYSDPIFGPKMRDFMEKCDFE
LFAAYICPRASPEALRTSMDITDAEDSVTVKKAEAIVVRTLQDRSFDDGSWICRLVKEFLD
LHVRKKAGPNVSRRFVGHFVDYVVRVSDEATQRERHEVLDIEAYVERRRESGAIRLTFDLI
ESGLHIDLPQYVHEDPAFIAGYNATMDLACWVNDVYSYNMEQAKGHEASNILTVLMKYE
HLDLQAAVEYVAKHCEVLAAQFIEAHANLLARSDPNFSEDAARVLDALGDAVIGNDRWS
FETERYFGKDYKAVKQSRIVKLAGRAEGKHALRN
>CpSTS9
MTVLQFVFSVFTSSKPQTFFLPRLDETFKVLPNNGLNPHFSTVRPQSRAWIKQYDGEVCGP
KMRAFMDNCNFELSNAYTYPYAEPAGLRAAMDLTNILWLYDEYTDTEDGASAERAAVIV
NRALKEPGFNDGSWICRMMQDFRRRHIDKAGPDVARRFITNFCNYVDRVAREAILREKNE
VLDIPSYILFRRETSAVKTCFDIVEYCLGLNLPQYVHDDPVFVSGYNAAMDLVFWANDLFS
YNMEQAKGHSGANIVTVIMKSKRVDLQTAADFVAGYCEALTAQLLEAKQILSSRSDPAYS
KDAVRVLEAFGDWVRGNDDWSFATERYFGKENNAVRESRIVVVRAPFDETVKLVE
>CpSTS11
MSQKSPSTFRIPDLEAIFSAFPDEGTSPYYDDVLPEARAWIKLYQDQVYGPKMTEFIDRCKI
ELITYYVHPVASRSCVRAMMDLHNLFWLYDEATDVQSGQTAQETAKVVRNSLTSPEFND
GSWLCEMLQDFRKRHLDGVRSSSFVARFIEHFCFYTDRVADEAIYREKQRVLDIPSYMAFR
RETAAVRVVMDTVEYCAELELPRTVLDDPVFQVAYDAALDLAFGTNDIHSYNMEQSKDH
SGANVITVIMKERGLDVQGAMDYFGGYCEALTAQFLDAKRKIEKREGQEWKDAVLILDG
YTHFLTGQVRWGFATERYFGKKNKEVQETRVVELRAPFVDHVDLAD
>CpSTS12
MESLPVTLVLPRLDDIFDDLPNNVVNPNYSIACPASRLWIEQYGTQIYGPKMQAFMNNCN
FELSTTYTYPYADAKRLRATMDLVNILWLYDEYTDRASGAHAKEMASIVYQALSGQQVA
ANSWVGCMMQDFYRQHIEKAGPNTSRRFVDHFCRYAQQVGEEASLREQRQILNMHEYID
FRRETSGVRSCFDLVEYCLGIDLPQFVHDDPIFTMGYNAAMDLVFWVNDLYSYNMEQAK
GHGAANVVTVIMKSKSLGLQAAVDHLADACEVLTAQFLEAKSRLSKHPESIFSKEAVKVL
DAYGDWVRGNEEWSFVTERYFGKENKAVRQSRMVKIKTPFGEMAPFSGRKNYL
>CpSTS13
MPQDPPTTFRIPDLETIFSVFPDEGTNPHYDDVFPEAREWILRCKVELLTCYIYPSASKSRLR
SMMDLYNLFWIYDETTDVQTGQEAKETAQVVRNALTNPDFSDDSWLCAILQDFRRRNLD
GIMSPGFVLRFIDHFCDYANGVADEALFREKDLMLDIQEYLKFRREAAAVRVVLDSVEYC
LDLELEQNVMDDPVFQMAYNAALDLAFGTNDIQSYNMEQAKQHKGANIISVIMRARTLD
LQGAMDYFGGYCQALTAQFLDAKCIIEKRVDRPEWKDAVRILEGYGCFLAGQVRWGFTT
ERYFGKKNKEVESTKVVELRAPFVDHVHISD
>CpSTS14

MNVMNTPITTATMPVSPPCPNPDPTFQTRLSARWHPSTAEIVPKVRQYIVQNWPWSSEESK
RRYLTGNAEDGVMYAFPGVKNDRIEAVTTWLALHFLIDDYILESVSDSAASGLDPGKKKE
AIQRLFGIMRRLVRPNVNNPVEVMVDHVASSFLSCSSEDEVAQRHARQILESTIQFITAAEN
GDGKATAMGDLNSYLAYRMVEAGVFLSLDLAFWGGEIYIPSHISNDPQIRSFFKLVSDHLV
LVNDIYSYKVEQDRSEGVGGAFNAVSVLMRSKHVDAQDAMGIIKTQLTTLEEATLEEAAV
LRDRYAEKGEEEEVVDKMIQTILEMMAGNCEWSKFCGRYNSSPVSDEASVGTDRALEQG
TTSLS
>CpSTS15
MSSHRPTQQPNPLVDPPDFVSKFLAHDREDRDEIITSVQAYFLAHWDWPSPTRKAWYSKA
DLEDWTTLMCPAGPSERAWAFTAYVTFWFLYDDLMEIMGPEELGSSIPRIARILHGEEGLE
DMTTPEFILKEICLKINTIAPGKRIFEATLVYMKAATAKQERLQSLTLGFDAYLKYRMLDAA
CWLTLEGAYWVQEITIPEHLKTEKIWLLQELSLFHGILINDLFSYRKEVKASSQEVQDHDK
TLYNGLVILMQSYQFTLLEALEEMKKKIWDYEDEFMAVLDDVRETHKSSAEDREFIERLA
VALMDVIGGNVAWSACCGRYNRL
>CpSTS16
MPLPVTFRLPDLSSIFSIFPDLGINSHYECTYPESREWIAQYHTGVYGPKMRDFMERGKIEL
LGAYTYPYASKERLRFVMDLHNVSWLFDESSDVKTGQQAGVTAVVFRRALIDPEFNDGS
WLCHMLKDFRQRHLDNVMSHAFVEMFIRDFIGYADGMSAEACYRDLNKVLDIAGYVKL
RREAGAVRLALHSVEYCLEKELPSYVREDPAFVIAYNGALDLGYIVNDIHSYNMEQSKGH
GHHAANIVTVFMEAQNIGLQAAMDYAGGFCHGLVLQILEAKELLAARSDPVFSNDAVKV
IEGCINFFKGQDIWDFESERFFGKSKDAVRKTKVVNLRALFEDSVNLNE
>CpSTS17
MNSDLQSTLASLDTTYTCRFRARYHAKSEQVMTVVQDYFVKRWPWRSEQAKQLYIKTN
LEEATCICFPTTLDDRIETVVTWFCYMHILDDIIEGLEANKATKLVDRLSNIILGTLDPDPED
RLGVMAADVCSRFRLGENDGEEKHGLDIISESCKLLRHTSSADSKLQSLSTYDTFIEWREL
DVGLWFSTSLFLWGCGIYMRLHMSNDPEVRQLLRVSGRHIAVANDLFSYRVEALRAGQTS
QILLNTIAIIAKEKQVDPQTAMNMTKQRLSEMEEEVEVLIGKLRDRYTGEEGELIERLFVV
CKGMMAGNCEWSSICFRYNGAQRAVAA
>CpSTS18
MVHWNHPPTFVLRNICDITGRVFELKENPRIAEANSAVLKWFEQFNVYDKKKADKFLNV
GKFDIFAALSFPEADLEHLTTCLIFFLWAFATDDLSDEGEFQSQPDQVQHGHDISCSILDDD
DAPQPDYPYAAMLWDLLRRLRSTGHMGMYKRFKQAFLDFSSSQVQQSTNRNVDRIPPVD
EFILMRRKTIGAALVEAMVEYSLDLDIPSYVWEHPVIVGMSQATSDIMTWPNDLCSFNKE
QADGDYQNLVCVLQHNHGLELQEAIDLLTKMISDRVQDYVDLKNQLPSFGPDVDPALHT
YLTALEQFVQGTVVWYYSSPRYFRHLDPRGKPEVLIHLFPKTDAPTLPVVVQEKSRPFIER
EIYLPAKRLLGVFVNYVAITVFGYSVYRLYGDS
>AcTPS4
MSAQQFTLPDLLAVCPLKDATNPHYAQAAAESTAWVKSYNIFDARKLAFLLQGSSELLVS
HAYPYAPYEQFRTCCDFVNLLFVVDEVSDDQNGKDARRTGEVYLNVMRYPDWDDGSAL
AKMTREFKQRLLAFAGPNSYRRFLMHCDDYVNAVAREAEYRERGEVLDIDAFQTLRREN
SAIRLCFGLFEFALGIDLPDFVFQDPHFMTLYWSAADMVCWSNDVYSYNMEQAKGHTGN
NIVTVLMRQKSIGLQEAADLVGAHFSALMGRFVETKKQLPSFGAAALDDAVAKYVAAME
HWVIGNLEWSFESQRYFGAEHTRVKATRVVVLSPADEN
>AcTPS5

MAVTPAPVNGSDSTKEIILKFPDFISPIPYPLRCHPQEREVSRQSEEWLLSMANFSEKQRAK
FLTLNAGLLSGWCYIDCTFDELRVCTDFMNFLFTLDDWTDEFDTTGTRGLAECVMNTLYF
PDTYKSDTAAHRLTKSFWERMRATAGPGCQQRLLSTLDTYFQAIMQQAADRGSRNIPDLE
EYILLRRDTSGCKTGFAFIEYAANIDLPDEVIEHPIIKAMSDSTNDLVSWANDVLSYNAEQS
RGDTHNLVCVLMHQNNVDRQEAIEQAGELWRKTLDYYFECHKALPSWGPEIDRAVALYV
QGLDDWIIANAEWSFETERYFGKEGPTVKKTRQIPLL
>AcTPS7
This sequence is not available.
>AcTPS9
MSSPSSFVLPDLHAVTPFKGSFNPHYPEAAAESSEWVNSYKVLSDKKRAFFLQGGSELLC
AHAYPYAGYQQFRTTCDFVNLLFTVDEISDDQNGKGAYETGLTFYNAMSNPAYDDGTVL
CKMTKEFTARLLEHCGPQTYRRFIKHCKDYIEAVAVEADLRERGEVLDLEAYQTLRRENS
AVRFCFGLAGYALGIDLPDEVVEHPAFMAMHLSTVDMVCWSNDLYSYNMEQAMGHTGN
NVITVLMQHKGLDLQGAADYTGVHFKGLIDTFLDAKRSLPSWGPKLDGEVAQYAMAME
TWVIGNLNWSFETQRYFGHARHEIKRTRVVQLYPRRIVEESSDEEDN
>Tps1A
MSPRYSALLKVLCRMPGSLDGFVSIFRRFMQDPLGIRYHLHTLLLFTWADMKTILLPITAF
ACSTAPLHSFSNLVQGMIWIWLHQLLCNVSNQARGKSEDALNKPWRPLPSGRLTEPQAVI
LRWITVAVCLLLSATYGRDLLMTTVGLILTTLLYDELGMASHHIGKNLCNIGGYTTIEVGAT
KLMGASRDLDYVSTVAVIISGVLIFTTIQAQDFPDIEGDAALGRVTFPIYAPEFSRIFTFIVMP
AWSIFLGWFWDIGVISRMVFAALGTYVGLRYYLWRTVDIDKRSYVFFNAWLTLAHILPLS
VRTGFLAF
>Tps2A
MASKRTFPVISIRPLLSLCFSALRNWIRTLILFTYTDYKTIVLPVSVFACVSAPVHSFVRFLH
AVLWIWLHLLQCNVSNQYRSVLEDAVNRPWRPLPSRLISVEHACILRWLLVPLCIGTSLCY
GWDVALASACLTLTTVCYDELGLAGHFLGKNLCNVPGYVSFEIGATKIMGSTTNLDFIALE
SILCSAMVIFTTIQTQDFPDVAGDRALGRVTLPILCPEGSRHFTTCVLLFWSGFLSYAWSIGL
LSSAVLISLGIWVAYRYYRFRKVEEDKKSYLIYNIWLLFVHSLAAHARWNLMAL
>AncA
MRRNVLNKATHSQSPLKPNITTLIFDLGDVLLTWSDSTPKSPLPPKIVKGILRSLTWFEYEK
GNLTESQTYGQVAQEFGVDASEVKASFEAARDSLKSNPMLLQLIRSLKDSGHVIYAMSNIS
APDWEFLKTRADLSDWALFDRVFPSAEAHDRKPNIGFYQHVINETGLNPSNTVFVDDRIE
NVVSARSAGMHGIVFDDINNVIRQLKNLCEDPIHRARSFLYANKKCLNTVSTDGTIVSENF
SQLLILEAIGDESLVDFVRHEGRFNFFQGEAKLIMTNHYPDDFDTTSIGLTVVPYIDDKTRN
RVMDEILAYQSEDGIVLVYFDHKRPRIDPVVCVNVLTLFYRYGRGHQLQKTLDWVEQVLI
NRACASGTFYYATEEQFLFFLSRLIQSSPDVRQRLEGVFKRRVVERFGADGDALAMAMRI
HTAASVGLVDHVDLDKLFALQQNDGSWRDSAFYRFPSARQLASNDGLTTAIAIQAIQAAE
RLREDGNVL
>AncC
MRRNVLNKATHPQSPLKPNITTLIFDLGDVLFTWSDSTPKSPLPPTIVKGILRSLTWFEYEK
GNLTESQTYGQVAQEFGVDASEVKASFEAARDSLKSNPMLLQLIRSLKDSGHAIYAMSNIS
APDWEFLKTRADLSDWALFDRVFPSAEAHDRKPNIGFYQYVINETGLSPPNTVFVDDKIE
NVLSARSAGMHGIVFDNINNVIRQLKNLCEDPIHRARSFLYANKKCLNTVSTDGTIVSENF
TQLLILEAIGDESLVDFVRHEGRFNFFQGEGKLIMTNYFPDDFDTTSIGLTVVPYIDEETRN

RVMDEILAYQNEDGIVQVYFDCKRPRIDPVVCVNVLTLFYRYGRGHQLQKTLDWVEQVL
INRACASGTLYYVTEEQFLFFLSRLIQSSPDVRKRLEGVFKRRVVERFGADGDALAMAMRI
HAAASVGLVDHIDLEKLFAMQQNDGSWKNSAFYKFPSARQLASNDGLTTAVAIQAIQAVE
RLGEDGNVL
>BvCS
MSTASSPSLVASEIDSPHHSRTSSPSPTLSPPTSFILPDLVSHCNFPLTYHPAGDEQAAASLAW
MLSFVPHFTPKKVAAMNGLQAGELTAYCYHDCPPERLRVVDDFMNYLFHLDNISDGMM
AKNTTQLADWVMNAFEWPEKFQPTVNADGEVVEEIAAVKLARDYWSRCIQQAKPGVQQ
RFKSSMNMFFQAVEQQTNDRDGQVVPDLESYIDMRRDTSGCKPVFDLIEYALGFELPEEV
VDHPVIKALNQDANDLVTWSNDVFSYNVEQARGDTHNMICIFMEHDGCTLQEAIDRVGG
LCKQTIDAFVENKARVPSFAHLGPEVDAWTTGYVQGLQDWIVGSLHWSFMTKRYFQEAG
AEVKKTRFVKLLPIEEGRHKHIPPIYASAMVAATA
>GME3634
MAVTPANVASPDSQEIVLKFPDFISPIPYPLRCHVQEREVSRQSEEWLLSMANFSEKQRSKF
LTLNGGLLSGMCYIDCTFDELRVCTDFMNFLFTLDDWTDEFDTTGTRGLAECVMNTLYW
PHSYQADTAAHRLTKSFWVRMKQTAGPGCQQRLMSTLDTYFQAIMQQAADRGSHNIPEL
EEYILLRRDTSGCKIGFAFIEYAANIDLPDDVIEHPIIKAMADATNDLVSWANCPFVQRTVT
RRHAQPRLCSHGSERPRPAGCDRAGGRAMGEDPPLVLRVPQERPFVGLRDRPCRRSVHPG
ARRLDYRERRVELRDGALLWQGRASREEDAAGYTASGPYSC
>GME3638
MRARSFILPDLVSDCPYTLRCNSNCEAVARASEAWMLEDANLSPKRRDAFLRLRGGELTA
ACYPDTDEACLRVAADFLNFLFSLDDWSDEFSMEDTCGLAQCVMCVLHDPDDFQTEKAA
GKLAKLFQSVPADGGAEVYSSIHRYGPLLSCDCTASPGPRVRVCSLTRRICGPPRGHERVQ
ALLRSHRIRRGYGPSRPRRPSSNNHRPRAGSQRVYIMVERSLLVQRRASAGHTQHDCGDH
ARGRAQLARSRILGRSLQALHSALRGKPSHAAIVGSGD*RGSRQVCARPPGLDGRCPPLE
LRHRTLFRGRPCDQEARCRDTTTAEVFI
>GME9210
MPLSSSVVAFRLPDTLGCWPWRRCLNTHYVEAKQDSASWLESFHPFGPKAQRAFNKCDF
TNCVQGVTTSSSSSTSTLMSRTRRRSSSSRTSSWTHCETLTSLVLQENLSWAKSLGSWARTI
KVASEPSQRRFIETFDDYCQSVVQQAADRSQNHLRDVESYLENRRENIGAKPSFALLELD
MNLPDEVIEHPTIVNLTTWAIDMIILGNHCLVQRGAGAWRRRTQRRDHRHASLQRRRAGC
HGPHRRVAPEAGGPVPHQLQQAAVVGTRDRASAVHPGHRKLGTRERRMEFREREVLWIE
RTRDRAESLGNPTPSGLRGEACGC
>Agr1
MCASATRPQPSASNNVKKIILPDLVSHCTFKLRHNRHRKQVTTETKKWLFKDGNLLGQKE
RAYHGLKCGLLTSMCYPDAGYPQLRVVNDFLTYLFHLDNLSDEMDNRGTTTTADEVLNS
LYHPHTWRSSARVGKMTRDFYKRLVLTASPGAQQRFIETFDFFFQSVTQQALDRASGVIPD
LESYISLRRDTSGCKPCWAMIEYANNLDIPDEVMDHPIIRSLGEATNDLVTWSNDIFSYSVE
QSKGHTHNMIPVVMYQEGLDLQAAVDFVGDMCRQSINRFVEEKARLPSWGPKIDQDVAI
YVQGLADWIVGSLHWSFETERYFGKSGRQVKASRIVDLLPRQRLP
>Agr2
MVWDFVLSLFHSLLAAFQTLTSWLTGSFLFNNKMAPAPNPAPVTFILPDLEKTFNSLPDDG
LNPHHDVACAESREWFAKYNKKVLGAQMQEFFRRCKFELITSYTYPYVDKEGLRATMD
WHNILWFFDEVTDTETGKDAHKSAIITIRTLREPDFDDGSSLCRMVRDFRLSHLSRAGPEC

TRRFLEHCDVAFHAGAVEAELREKGEVLSIEGYLKLRRETSGARTCFDMAEYLMDIDLPQ
DMYDDPVFQKGYIAALDLIFLANDLYSYNMEQAKGHNGANVLTVVMKETKLNLQSAAD
YVGVLCEKLIKQFQEAKSTLENRLAKEKNPAKAAALKDAIRSLVGYGHWVRGNVEWSFE
TERYFGKKNKEIKKSRVVTLTPTNSVNRALKA
>Agr3
MNASPFLNESSPTRPTSFVLPDLVSHCKFPLSYHPNGDEIAQESVDWLDSSCPDLTAKQRR
ALRVLQSGELTAYCYNQATSPERLRVVSDFLTYLFHLDNISDGMMTRETDVLADVVMNAF
WFTDKYMPTRGPGKEQLDEELNPGKLARDFWSRAIADCGVGVQARFKETMGLFFEAVNI
QARMRDEDTIPDLESYIDVRRDTSGCKPSWVLIEYALGIDLPDHVVDHPIMQALNQGTND
LVTWSNDIFSYNVEQSRGDTHNMIVILMEYHGHTLQSAVDYVGELCAQTIDTFCENKERL
PSWGPEIDDMVARYVKGLQDWIVGSLHWSFQTQRYFGKDGLDIKKHRFVKLLPLEAAK
>Agr4
MSALPSQFKLPDLLSTCPLKDGTNPAYKKAAAESRAWIGSYNMFADRKRAFFIQGQNELL
CSHVYCYAGYEQLRTTCDFVNLLFVVDEVSDEQSGEDARATGQVFVNAMKYADWHDGS
KLAKLTKDFRVRFLRLAGPKNVARFVALCESYTACVGKEAELRESGQVLGVKEFIPLRRQ
NSAVLLCFSLVEYILGIDLDDEVYRDENFLNAYWAACDHVCWANDVYSYDMEQSKGLSN
NNIVTVLMEENHTSLQDTSDYIGEKCAEFVQIYLTSKKRLSPSLGPDAALFLESIGSWMVG
NLAWSFETSRYFGSRHLEVKETGIVILRPRELPEDGSSSDSDEE
>Agr5
MASSLLEPSLAAIALVILLASVSLSRKKRPAAPEPQGLSVLGNLFDIPKRASSIIYLALGKPY
NTLTKRAVSQLQGYTPGSHIDATSHSPRVFRLPNLEETFSVFPDHGLNPNYTSARTDSRAWI
NQYTKVVCGPKMVAFMNNCEFELSNSHCYPYAGYKGLKATMDLTNILWLYDEYTDTGSG
AEAVKAAGIVARALREPDYDDGTWVCRMMKSFKQNHIDKAGPGVARRFIDNFCNYVEV
VGREAELREKNEVLDIPNYVTFRRETSAVRTCFDLVEYCLDLDLPQYVHDDPVFISGYNA
GMDLVFWANDLVSYNMEQSKGHSGANVVTVIMKSKGVDLQTAVDFLGGYCEALTAQLL
EAKRILQARSDAAYSRDVVRLMDAFGDWVRGNVAWSFETERYFGKENKRVKETLLVELK
EPFVGALALKE
>Agr6
MPGSANWTADRFYIPDTLANWPWPRAINPAYEECKAASAAWCEKYGAFSARAQKAFNL
CDFNLLASLAYAGLPADVNRVGCDLMNLFFVVDEHTDAMDARSVQDWVDIVVDALHHP
HTPRPAGEPKVGEIARTFWENGIKCMGPTAQRRFVETFTTYLQSVVTQAQDRDKHLFRDV
DSYMEVRRDTIGAKPSFALLEHDMELPDDVFYHPLLEKLREWAIDMLILGNDLCSYNVEQ
SRGDDGHNIIRLAMLQENTNVHGALRFVSKMHDDLAEKFLSNYQGMPSFTPQIDAWVTR
YIDGLGNWVRANDSWSFESWRYFKGDVLRVQAERWVELLPPAPKDELTPA
>Agr7
MSFFKSSQPTIYIPDTLRNWPWPREINPHYEECKRESAAWVEKFGAFSAKAQKAFNKCDF
NLLASLAWSRVNRDGCRIGCDLMNLFFVFDEWSDVSDAEETRRMADIIMDALYDPHKPR
PTGEWVGGEVTRQYWLNAIRTATPSAQKRFIKAFKLYTDSVVQQSADRDKHLIRDIDSYF
EVRRDTIGAKPSFAINEVHMNLPDYVMEHPVIKNLTAYCIDMLCIGNDLCSYNVEQSRGD
DGHNLVTIVMHQLNLDVQGAFDWIGKLHDELVDKFLEEYKNVPTFKDKQVTKECAEYAF
GLGNWVRGNDQWSFESERYFRKDGMRVLTERTVVLLPKKREPPPKPLDEDPIYSALPWW
GWTALFGFLATAFTFSARQLSTRISANLIA
>Agr8
MSEQQYTLPDLLQNWPWNRHLSPYYEEAKRESSAWVESFKPFDQDGQRAFDAYLLASLT

YSHGSREFVRLGCDLMNFYFVYDEYTDVSDSAVADRLANIVIDAMRNPENSSQSGDHLLG
KMTKHFWTRALAMAPAGSPCFEHFITTSETYLRAVTQEAEDRANKRVRKVDDYLRLRRD
TCGARPTLALIEFGLNLPNEVVRHPSLVALTEAAVDLIILVNDMHSYVRELSCGHENHNLIT
AIMLEHRLNRQDAFHWLGSHCSRVVDQFLSDLDELPSWGEPTDSGVRDYINGLGQWVRG
NDDWSTESKRYYGEDGETIRQERLVTTRSGESNYIKFGQVGVQDSVRIQPIEAN
>Agr9
MTAAPLTFTLPDLLANFPWKRNLSEYYPECKTESSAWTESFHPFDDEGLKGFNLCDFNLL
ASLAYSPREREIIRLGCDLMNIFYVFDEYTDIADGDGADKIRDIIMDAFRNPHKPRPEGELL
VGEMARDFWIRASGYVSPDAHCLTHFLRDFDTYTAAVVREADDRAKRVYRTFEDYLSIRR
DSSGCLPSFALCEFGLDLPEEAYHHPRMAALREQSTDLIAIGNDIDSYAMEKARGLELHNS
VELIINEHGLDVQGAINWLERYAAGVHASFLDNVANMPSWGEDVDRRVKMYIDGLAQW
VRGNDDWTFESGRYFGDKGLEVQKTRVMSLLPASKVSLRSRPKAVGHVPKKLLRYFRYS
TMYFFGFHVLAK
>Agr10
MSLNFFLRRYTVFPWSRKLGQYYHDAKRESSAWTESFHPFDEDLSKAALTFCITALLASLA
YFLRQKEIVRLGCDLMNIFYVFDECADIADKEGASQIRDVVMDDLHRPEKTCPGGEILPG
EMVKYVLLLCPEIPETYYNTKSFGFAPQSSSPQPHIVCATLSRISMPTQQQWFNVKRTIGPN
VFLARSATVLPYAETHTFYHPRMIALREQAPFLDINSYPMKVRGLVQGSINWLEGYAAGV
QAAAFLDNIANLPSCAKEVESRVNIYVNELAQWARGNDDWTFESGRYFGDRGPENQSDIP
TSSNR
>Agr11
MQIILPDILQTWAYARLLNPHYDGAKLESSLWIHPLVAKLFDQKGQKAFQNDYTSLLASL
MYSHQGKVPSRRCDMMNLFFVYDEYTDVVSPEIAHRLSKIVVDAMKNSDEMSPCGEHPI
GDKAKEFWRLATTLLPATGSNSDVCKSRFINLTEEYLNAVTVEARDRNEGTIHSVKEYLTM
RRATSGAGLMLALIEFELDLPKAVLEHKFVQALEEIYTRTVSSGQANHNLITVVMHENPGL
SLQGAFDWLGSYAAGVVECFQTNVRNLPSFCDVEGPACESVDGTLQERVDKYISGLGQA
VRAEDDWAFETTRYYGEDGPKVRETRVLVIRPVKRITRRHLLQSLEIKYSMVRG
>Copu1
MGLPQATEFVLPDFFAPCPFTLGLTNPHADTVFPEARAWIGKFLPFSGLAWHCDPWAGKE
GFRTICDFQNLLFLMDELTDEMSGEDARVVGESFIRVLKDPSVDNESIIAQATREFRTRIAD
DVATKSVWFGRFVAICKSYADATYIEAEHRENNRVLDLDDFVIARRENSAVRCCFSINEHA
LGIDLPDSVFEDPEFLRMYFDAVDMIVIVNDVYSYNMEQAKGLAGNNVVTVLEQALGVD
LQAAVNRGGEMFAQKMEGYVRGRGVPPSWGAKVDADVEHFFDSVDQWIVGNLEWSTE
TSRYLGPDHEEIMKTGRVVLRKIESKTK
>Copu2
MSTMDPSEFILPDFFATCPFAFGRTNPHADVVIPEAHAWIVKHVPFVDRKRDEFIQDGFQD
LMPHCYPWAGKETLRTMCYLNNLLFLVDDLTDDMNSDEARGFGESFIRVLNDPAVHDSSQ
VMQATREFRSRITGTGVTESRWFGRFLAIFKLYINAVCAEAEDRENKRILDLDTFTVARRE
NSAVMVFFAITEYALGIDLPDAVYEDPTFLRVYADSADMVILVNDVFSYNREQAKGLDGN
NNITVLMQTLDLDLQAAVDHVGEMFSQKMEGCMRGRAMLPSWGVKVDADVERFFDAL
DQWVVGNLEWSSQSPRYLGPEHEEIMRTRRVVLRKVETEIE
>Copu3
MSATPAPTEFILPNLFSVCPLTFGRSNPYYDEVIPEARAWIAKYNPFVDSKRAEFVQGCNEL
LCSRVWPYAGREEFRTCCDFVNLLFVLDELSDDMGGADARSTCDSFIRVLNDPDAPDTSLI

AQMTREFRARVAERAKPGCLRRFIALCGTYVEAVCVEAELREQGRVLDLRSFILLRRENSA
VRCCLALAEYALGLELPDAVFNDPAFQSVYFCAADMVCWSNDVYSYNMEQAKGHTGNN
VVTVLMQEHGIDLQAAADRVGEVFGQLMEHYTSGSRSLPTWGGKVDADAARFLEAAGQ
WVVGNLEWSFETPRYFGPDHDEVRDTHRVLLK
>Copu5
MHLPEPFHFLLPDFSSHCSYPLRLNKHCVAAAAASEDWLIRLAQLRSPRNGRKLKKFMGL
KAGYLTALCYPDCPRTELRVVSDYMNFLFTLDDWSDEFAEAGVRGLEQCVMGMLYDPTV
KTDKAAGRLARSFWLRMIRTAGPRVQHRFIVAFEDFFRAVEQQSRDRAKGVMPDLESYIA
LRRDTSGCRPVFVLAEYAAGIELPDEVFEHPIIQSMTEATNDLVTWSNDVFSYNKEQALGD
THNMITLLMAQHGLSLQGAVDFVGQLCAASITRFESGRTTLPSWGPDVDCDVQKYVMGL
QDWIAGSLHWSFETERYFGKRGKEVRQAGVVKLSPMKAPKKV
>Copu9
MSPTATFTTTSSEENAPTKFILPDLVSDCTYPLLLNDNCEPVARASEQWLIAGARLQEPRRT
KFMGLAGELTACYPHADASHLRVCVDFMNWLFNMDDWLDDFDVDDTWGMRHCCLGA
FRDPVGFETDKLGGLMSKSFFSRFRQDGGPGCTERFIHTMDLFIAVAQQAGDRANGITPDL
ESYITVRRDTSGCKPCFALIEYAAGIDLPDHVIYHPTLAAMEEATNDLVTWSNDIFSYNKE
QVTDDTHNMIPVLMRERGLDLQGAVDFVGRLCKGTIERFETERARLPSWGPELDAQVQT
YIEGLQNWIVGSLHWSFDSHRYFGKDGHAVKKHRIVKLLPKRVPQQA
>Pilcr_825684
MPSQSLTIRLPKFEETFSVFPDNGLNPHYANSRAESRAWINQYHHAVCGPNMRTFMDKCN
FELAGALFYPYANEAGLRATMDLVNLLWLYDELTDTKTETEAVNAAHIVACALREPDFDD
GTWICSMIKDFNQRHISKAGPNTAYRFIYNFCNYVEAVGTEAGLRAKNEILDITTYISFRRE
TSALRLTFDLVQYCLGIDLPQYVHDDPVFASGYNAAMDLVCWTNDLFSYNREQAKGHAG
ANVVTVIMKSKGVDIQSAVDFVGGYCEALTSQLVEARRILLSRSHRVYSKDAVRILEAFGD
FVRGNDQWSFASERYFGQKNKVVKESRIVEIITPFSDLIAINE
>Galma_104215
MNTTTRTFYLPRLEDTFSVFPDNGLNPHYAECRIQSQAWIDKYYKIVCGPKMRAYMDHCK
FELITAYTYPYASSDGLRKTMDLANILWLYDEFTDTLSGKDATNAAAIVIRTLRERDFDDGS
WICHMMRDFYAAHIEKFGPNVSRRFIDHFCQYVEGTGTEAKHREKDHVLDINAYIIMRRA
ASAVLTAFDLAEYCLGIDLPQYVHDDPAFISGYNAGLDLVFLDNDLFSYDMEQAKGHCTT
NIITVVMKSKRIDLQSAFDFTAGYCESLTQQLIAAQISLASRTDPVFSNNAVKCLEAIANWV
KGSDGWSFATERYFGKQNVIVKETRAVEMRKSFQDIAVLKE
>Sphst_47084
MPLLSCSQTFRLPPLHETFSVFPDNGLNPNYNACRAQSRAWISKYNVQVCGPKMRAFMD
NCNFELSNAYVVYPYAQPAGLRATMDLANILWLYDEYTDMQTGEDAAKAAVTVSKTLLNP
EYDDDTWICHMMRDFYVNHIQKCRPNVAHRFIENFCRYTEVVGTEAKLREKNEVLDIPG
YVALRREISAVRTCFDLVEYCLDLDFPDYVHKDPIFVIGYNAAMDLVFWANDLFSYNSEQ
AKGHAAANVVTVIMTSKKMNLQSTVDFIAGFCEALTFQLLDAKRALSLHEDPTFSRDAVR
CLEAFGDWVRGNDAWSFATTRYFGPENKIVKETRIVKLKAPVEESVALKE
>Denbi1_816208
MASKSPSPRTFYLPRLEDTFSVFPDNGLNPNFAAVRPESRAWINQYTKLVCGPKMCAFMD
NCNFELSNAYCYPYAEKPGLRASMDLANILWLYDEFTDTESGAEAQRAAIIVHRTLREPDF
DDGSWICHMMRDFRIHHVNKAGPNVARRFIDNFCSYVEVVGTEAILRERKQVLDIPGYVK
FRRETSAVRACFDLVEYSLGIDLPQHVHDDPVFISGYNAAMDLVFWANDLFSYNMEQAK

GHGGANVVTVIMKSKGMDIQSTVNFLAGYCEALTAQLLESRRILASRPDPVFNKDAVRVI
DAFGDWVRGNDQWSFATERYFGKDNETIKKTRIVEIKEPFMDSLALNE
>Denbi1_659367
MTVPIPTSTEQSSAPTRFFIPDTLANWPWPRALNPAYEQCKADSAAWCEKYKAFSPKAQK
AFNLCDFNLLASLAYAHLPEDVNRVGCDLMNLFFVVDEHSDAMDKNSVHVWVEIIMDAL
RNPTKPRPDDEPIVGEISRTFWENAIKCLGPTSQKRFIETFETYLYAVIVQADDRDHHVFRD
VDSYMVVRRDTIGAKPSFALLEHNMDLPDDVFNHPLLEDLRTWCIDMLILGNDLCSYNV
EQSRGDDGHNIVKLVMLQENIDLHGAMQYISDMHDDLADKFLRNYKNMPSWGQPIDEW
VTRYIEGLGNWVRANDAWSFESWRYFKYDGLRIQKERWVELLPPANKEDLTSSSE
>Hetan2_454193
MAQKIYIPDTLANWKWPPHLNPHYPEVKRESAAWLASFGAFSPKAQDAFDRCDFNLLAS
FAYPLAKKEHLRSGCDLMNLFFVIDEYSDVAEADEVQRQADIVMDALRNPHKPRPKGEW
VGGEVTRQFWELAIKTASPQSQKRFIATFDTYTQSVVQQAADRTHSYIRDIKSYFEVRRNT
IGAKPSFALLELEMDLPDKVIEHPIIQDLSILTIDMLHLGNDIASYNLEQARGDDSHNIVTIA
MNQLKTDVAGAMKWVDNHHKELERKFNESFEKLPKWGEPIDSQVARYVDGLGNWVRA
NDQWSFVSERYFGKKGPEIMKSRWVTLLPKERTEDIGPQVVDDSLL
>Hypsu1_138665
MALSNSNSTTATVTLPDTLRFWPWQRHINPHYSACKKASSEWCESFKAFSPQAQRAFNKC
DFNGCRIGCDLMNLFFIIDEHTDIASAETARTQANIIMEAIRDPEMPRSENEWVGGKAAQQ
FWLNATKSATPSAHRRFIDAFQMYMDAVVQQAADRSKNYVRDIDDYFVVRRDTIGAKPS
FAICELYLNLPDSVMEHPVIMKLTELCIDVIIIGNDLCSYKVEHEHGDDGHNLITVVMNQF
KITPQEAMNYISDLHDKLAVQFLDEWKNIPTFGGPLDLEVRTYCHGLGNWVRANDSWSF
ESERYFGKRGIEIQTTRQIEMNIQSHL
>Pro1
MSQRIFLPDTLANWQWPRHLNPHYAEVKKASAAWAKSFRAFQTKAQEAFDRCDFNLLAS
FAYPLADEARLRSGCDLMNLFFVIDEYSDVSTEEEVRAQKDIVMDAIRNTEKPRPAGEWIG
GEVSRQFWDLAKKTASTQAQKRFIDTFDEYLESVVQQAADRNNSHVRGIESYLEVRRNTI
GAKPSFALLEFDMQLPDEVINHPVIKELEKSCIDMLCLGNDVVSYNLEQARDDDGHNIVTI
AMNELRTDVAGAMIWVDEYHKQLESRFMENFKKVPRWGGPIDLQVARYCDGLGNWVR
ANDQWSFESERYFGKKGPEIIQRRWITLMPKMVSEELGPQIVDGFHL
>Hfas94a
MSAQQYIIPDLLANWPWQRVSNSMLDEVRDEANEWVMSLGLFEPAQFKKFRACDFNLLA
SFIGPLESKEHLRVACDLMNFYFAFDEYTDVASREEAMKIAQGVMDAFKTRSAEPSSSKIT
EMARQFFRRTVDVVGEDSPAINQFITDFDTYTTAVIQEADDRAEGTIRNVADYFTLRRETC
GAKPSFSFFALGLNMPTEVFEHPLIMSTVECATDLIAIVNDMHSYGLERARGLDGHNVVTS
IMYEHQLDLQGALYWLAGYAEDTIAKFFSEKERLPSWGRAVDLSVQEFVDRLGRCVRGY
DAWSYETNRYYGNCGVQIRQTRRITLPGNDSGYITKKEMGVSIA
>Hfas94b
MSYAPTVEELKQWEKGPNPELLFGNSFPLARDHPDWQKLEQENDQWVEENWQFDDKEL
REYLMSSKLGPFSSMCFPFGEREKLLWICRLVTLLFCLDEDLDRHVNLHLLPILKALTAGS
RKPENNYAELAIDKCWRAIERTSTPSTFRQFVQITHEYFDSHGQIPYENFDQYVGARRTNV
GAYFMWACLRYAAGIDLTDEELAHPLIKRLEDIAGFHIAFTNDLISYTKEFLTDTATNNILTL
LQRNDGLTPAEAEAKIRRELKKSEDDYQVAAKEVLNHPVLGKNKAIRKLVLNIPYGMGG
NAWWSLITKRYNVDPVNHPLPQVKIHIDPSMPYHPDAVAFLDNGSKKALTTKGAKSFLSS

WTVWFGALL
>Hfas255
This sequence is not available.
>Hfas344
MSQSQFTIPDLLASWPWPRAKNPALDQNLEDEANAWVASLELFEPRQLDKFKACQFNLL
ASLVGPIEGRDSLRISCDLMNFYFAFDEYTDVVSGDEVMMIVADVIQAFRDRESPEGSSKI
KEMARQFFQRTIALVGEDTQGIDHFIADFEAYAKSVVQEADDRVQGIVRNVEEYFILRRDT
CGGKPSFSFFGLGLCIPKEVFDHPVMQSLTESATDLIAMINDMHSYALEHARGLDGHNVIT
AIMHEHSVDLQGAFYWLSGHASKTVSKFLNDRKNLPSWGSDIDKAVNEYIDRMARCVRG
YDAWSYETNRYYGKNGLEVQKSRKIMLQHRELEMGYITRDQLLIGAA
>Cun3817
MVMYKRNATFNDGPVTQIQDIPSISSLFFTMVNQLRPFPSRFVLRDLTAVTEPVFKLRTNPH
QEEAYRNIERWFKDLKVYPEPKQRKELSHAEDLYAGLSFPDADVEHLETCIAFFLWAFSFD
DLSDEGEFQSKPDRVQVGVDISMEVLNHPELPPPKFKYAAMLHDVWRRFRSTASPGACD
RFKAVESWMKSQVEQAANRSWNTVPSVDEFILLRRRTIGGEIVEAMVEYSLDIKIPEYVW
DHPVLVGLSRAAIDIMIWNDLCSFNKEQADNDFQNLVFCVMLERNVDLQSALDIVTKML
ATRVDDYAKFKAQLPSEGAEVDQELAAYEKALEHYVQGTVWYYESPRYFRGMDVTDKT
DMVIPVYSRSADAPTSPSVSTRVSYLSNRSMPPRSSKASV
>Cun5155
MANESLTTTQLFIPDVLRSWPWPRQINPHYAICKAESDTWAQSFNAFSMKAQQAFLRCDF
DLLASLGCPRLDKEGCRICCDLMQLFFIFDEHSDIVDTTVVRRQADSIMAAIRDPTRPREG
EWIGGEISRQETANAMRVSTPTFQERFIVAFQEYTDSVVQQALDRDRHNIPNIEQYFNYRR
DTIGVKPSVAMLEIQFDIPNEVSNHPAISTLRSTCVDMIAINDLFSYNVEQALGDDEHNLTT
VVMHEHECSLTDALEWISDLHDSIANTFLSVMKTVPSFGDLVIDEQVAIYVDGLGNNVRA
NEANSFESERYFGKNGLEIQESRVVDLLPKQEN
>Cun3157
MSSNAVSFTLPDLLAICPLQGRTNPHYEAAAAESSANVLSENVFSNRKQDFFVSGGSELLC
SHAYPYAGHEELRICCDEVNLLFTYDEISDEQNGQDAYKIGLVLLKSLRDPEYNDGSVLCT
MTKQFQERLFPRMGPACYERFVDHVENYINAFVKHAEYREKNVVLDMASYEILRRLNSA
VRCCFGLFGYVLGLDLPDEIFEHPDMMAMHLAAVDMVCWNDIYSYNMEQAMGHTTNNI
MTVLMKAKNVDLQGAADYVGEYFKVLMDRFFDHKSKLPFFGPDMDPTAEQFVMAMES
WIVGNLANSFETLRYFGEKREQVKVTLVVELASKKV
>Cun3158
MPALTRTFKLPDLLSMCPVKGSTNPHYEKAAAESSANINSYNLETDQKRAFFIQGSNELLV
SHTYPYADYEQERICCDEVNLLFVYDEVSDDQSGRDARATGNVFLQVMRHDDWDNGSP
LAQMTKEFKARFRKFAGPGCYARFLVHCENYINAVGHEAEYRERGYVLDMESYETLRRL
NSAIRLCFGLFEFVLGVDLPDEVFDDPAFLNLYNAAADMVCWNDVYSYNMEQAKGHSG
NNILTVLMQAKNIDLQTASDLVGDHFAELMRRFLEGKRALPSNGLATDTAVAAYVKALEH
NVKGNLVNSFETQRYFGPKHEEIKKTLLVVLRAPSFD
>Cun0773
MVAHTFSLPDFFAKTPYQSRLHPLTGIVVQKSQEWVLRKVNYDEKERTAFLKTSGGLLCG
YCYPNADAFHIQVCADRMDWVFCLDDWSDECSVAEAQSVINSIKEYPRYPHEHSGSTPIIE
LAKNLYDRFFQTAAPGCAERFVRSMDIYLDGVVEQADCREKGSILDTESYTILRRKTSGV
WPCFALIEFAARIDLPDAVVEHPLIRSMEEATNDWISWNDILSYSKEQADKDAHNLIAVIM

NQYTLDLQSAVDLAGSHCFDCITKFEDNRKALPSWGEEIDREVDLYVQGLQDWIIGSLHW
SFACRRYFGAEGKEIKEHRTVFLLSEKHQS
>Cun7050
MQSATAPVLSSQPSKVVIPDLVSHCDFTLRCNSNQERASAECKEWLFQGSNLSERKQNAE
HGLKAGLLTSWCYPDAEYHHLRVCCDEVNWLFHLDNISDDMNTENGTSRTAVDIMNTLY
HPYSYSPVSPEGKLIQDFWRRLIPTASPGSQQRFNRTMDFFFQAVTQQALDRANGVVPDL
DSYIALRRDTSGCKPCWALIEFAYNLDLPNEVMEHPTIVALGEATNDLVTWNDIFSYNVEQ
SKGDTHNMIVVMMRQEGLDLQSAVDFVGDLCKQSIDRFIEQRAHLPSWGPDIDQQVEKY
VGGLADWIVGSLHWSFESERYFGKSGLDVKKTRVVELLPRRA
>Cun0716
MSRFNADSFVLPDLVSHCTYPLNLNTNWHSVSRASEQEVLEEANFSEMKRGVFMGLKAG
ELTSACYPYCDAFHLQVAADFLGYLFTLDDWSDDFDELGTKGLAVCVMNALRDPHGFQT
DKPAGILAKDFFSRYISKGGPGCIERFIDTMDLFFIAVERQAIDRENGVVPELEAYIPTRRDT
SGCKPCFALIEFAADIDLPDEVVEHPTIAALEEATNDLITWNDIFSFNVEQSRGDTHNMIIVA
MRERGLDLQDAVDFVGELCKQSIDRFEHDRLTVPSWGPEIDRDVRTYIQGLQDWIVGSLH
NWSFDTTRYFGQDGTEVKLHRQIKLLPRKGPCSD
>Cun0759
MARSTEALPDLVSHCPYPLRINPLCDIVTQKSEEWILNEAKYTPEKRIRFLNTKAGILTAYC
YPDADDFHLQVSSDYLTWLFCFDDWSDEFDETDACSFADCIMGCLRDPYGFKTDKAAGR
LTKSYFGRYLQTSGPRCAERFIDTMDLYLKSVAQQAADRDDGRTPDLETYIGLRRDTSAC
RPCFALMEFVAGIDLPDEVAEHPLIRSMEDATNDLVSWNDIFSYNKEQSRGDTHNLVAVIM
EERKLDLQSAMDFAGELCHQSISKFEADRRSLPSNGKEIDRDVQLYVQGLQDWIVGSLHW
SFATKRYFGTEGEAVKHHRTIQLLPRKDGVEPKDTVVRQNVIVYLFNMFFTVLVGSLFGN
MKEGLSSPLRVPRP
>Cun3574
MSPKRFVFPDLVSHCPYQLRIHPDCDTVNKTSEEWIMKDITFTPDRLKRFLDIKAGVLTAY
CYCDADLFRLQASSDYLTWLFCYDDWTDEFDEDESCSFKDCILGCLRDPYGYKTDKIAG
RLIADFFRRELQTSGPHCAQRFIDTMDLYLDSVGRQAADRIEERTPDLESYIALRRDTSAC
KTCFALMEFANGLDLPDEVSEHPLIREMEDATNDLVSWNDIFSYDKEILIEDTHNIVAVIME
DKKLNLQSAVDYAGDLCNNCIARFDEARKQLPSWGPEIDREVQLYGQGLQDWIVGSLHW
SFVSKRYFGLEGEAIKKHRTIELTPAMKSDVEA
>Cun9106
MTSMPQASSSRDPLELERLADELAERELRSPNAQSFQDHTLPAWAPLAHNNPYLSAETFN
VEEFLLSRSYTSLPDMRAELRDYLAILKEELVRLINDDYEAFISLSTDLRGEGTRLEKLKFP
LEELRSEVITSRTELQQIQDAVQLKLQRRSSLREEKAFLHLLLKISESVTRLESLLLIAAPSEE
EQHSTDFSVGTLQASRRGGQHEDEDRSRTNRAKHLSRVAAEYTQLLYHVNKAKIDQCAF
VDECQWRIDRIRSTLSSDLDHLFSTTLKALTRGKDHKPSTESEKAKLMADVSECLRTYDSL
GLWRDAEEVLRRDVVHDFVKKVYIMLTCKIIMSLTVLIPDNSCQFANSSPQSNNAAYTLPS
YSPTQPQYYT
>PpSTS01
MSSSAPSTSAPTKIVIPDLVSHCTIPVRCNRHWKQASVESKRWLFRGGNLSDRKRDAFHGL
KAGYLTSMCYPLAGYPQLRVSCDFMNYLFHLDNISDEMNDRGTHGTAVSVLDALYQPHM
HPTSRVGKMTKDYWVRLIQTASPGAQQRFIETFDMFFQAVTQQAMDRANGVIPDLESYIA
IRRDTSGCKPCWALIEYANNLDLPWEIMDHPIIRGLGEAANDLVTWSNDIFSYNVEQSKGD

THNMIVVVQNQQGLDLQSAVNFVGDLCKQSIDRFHYLRENLPSWGPELDREVEIYVDGL
ADWITGSLKWSFESERYFGKAGLEVKKTRVVALLPRRA

>PpSTS03

MGSISSTPSQKSPVFPARSLLPSDIVAVRPEGDEAKVLKFPDLVKSIPFPLRLNPYIRFVSAES
DAFIIEYANFSEKQRNRFIGLNAGLLCGMCYAECGPEQLRVCCDFMSFLFNLDDWSDEFDT
AGTKGLEEAVMNTLYHPDTYVSDTVAARTARSWWTRMLKTVGPRCRQRFVETLGFYFK
AILQQAADRSSKTIPDLETYISLRRDTSGCKTGFALIEYAAGIDLPNEVVDHPIIQSLLDATN
DCVSWANDILSYNREQSRGDTHNLVPVIMQTVGIDRQAAIDYAGDLCNKSVAHFLEGKA
ALPSWGKEVDVQVEQYVQGLEDWIIANAEWSFMTERYFGKDGPKIRKGLQVSLLPVVGF
D

>PpSTS06

MTVIADTSRCFILPDLISYCQFPLRCNPHRDAAQSSTSWLINNYPGMSPEQLVEVRRLDAD
TLASYCYPDCDVERLRVASDFLAILFHLDDITDTMEEGGTEQLEGTIMDAFRSEGKLDQRE
DEPRVRVPAKDLWTRFIRNAKPCVQTRLRDNIALFFKTAREEARDRERGVLLDLESYINMR
RGTSACLSCFALTEYSIGIELPQYVVDDPIVQALNQSANDLVSWSNDIYSFNNEQAHGIHN
MIVILMKSQGLGMQDAIDYVSDLFKQTIDGFMENTQLLPSWGAAVDADVRLYVQGLQD
WVVGNLHWSFATERYFGKRGAEIKATRVVELLPKKPVS

>PpSTS08

MLYLPDTMSAWPWQRAINPYFNEVKAASNSWFKSFRAFSPASQKAFDKCDFCLLAALAY
PRARKEHLRTGCDLMNLFFVIDEYTDVEDANVCRDMVDIVIDALRRPHDPRPEGEVVLGE
IARQFWARAIETASPTSQRRFLETFIAYLESVVLQAADRDCDAEHTVQTYLAQRRDNIGSY
PSYAVLELALDIPDDIFYHPAMNELSLYATEMLIIDNDLVSYNREQASGDTNNILFVIMRQF
NCSLDHAMAWAAAYHSQLEARFMDAFKRMPSWGLEIDSQVEEYCQGIANWPRGNDCW
SFESGRYFGDKGREVQKTRCVPLLPKKERDTSLRQQDVVITSL

>PpSTS09

MVRTRPTYIYLPDTAAGWPFPRTVNPYYEETKAESEAWISSLYPFDAYVQKKFNACDFTLL
ASMAYPWLSKDHIRTGADLMMLFFVFDEYSDVASVKDAQEMVDIVMDALRNPHKPRPK
DENILGEIAKQFWERGVKTASAPSARRFVDYFEGYLKSVVEQAQDREHNRIRSIAEYFDV
RRLTVGARPSYALMELGMNIPDEVWEDPAMEIMAVCVTDMIILDNDMLSWNVEQSRGDD
AHNIVRIVMEANKTDVASAMKWVEDYHNLLKKTFLDVYNSVPSWGPEVDAQVQEYARG
LGNWVICNISWSFESARYFGKEGRRIREERVVAILDKPVLVGVLESDA

>PpSTS10

MPSTPRQFVLPDLFPLVPFKGSTNPHYVKAAAESSAWINSYNVFTDRKRAFFIQGSNELLV
SHTYPYAGYEQFRTCCDFVNLLFVVDEVSDEQNGKDARHTGNVYLKAMRDPEWNDGSV
LAKMTKEFRARLLQYAGPGCYARFLKHCEDYVEAVAKEAEYRECGVVLDMASFETLRRE
NSAIRLCFGLFEYCLGVDLPEYVFEDPTFMTLYWAAADMVCWSNDVYSYNMEQAKGIG
GNNIVTVLMQAKGIDVQAACDAVGEHCKLLMERYLDAKEKLPSWGPSVDDAVAGYVQA
MEHWIIGNLEWSFETQRYFGAVHAEVKATRVVMLRPREIDED

>PpSTS14

MSDQPKMIYLPETMANWPWPRYINPHYEEVKAESDAWFKGFKPFTKQSQVAFDKCDFGR
LASLAYPWASKEHLRTGCDLMNVFFMIDEYTDVECASVVRGMVDIVIDVINNPHKPRPEG
ESLLGEITRQFWERAIKAATPSSQKHFIEAFTDYLNSVVEQAADRDNNHIRTVDSYLKTRR
ENIGARPSYFPAELGLNLPDEAFYHPVVTELSYNIAELIILDNDIASYNKEQATGDDRHNILT
IVMLQFNIDLEAAMTWVASYHKDVENKFLDGMKKLPSFGPVVDKELEEYILALAIWPRT

NDCWNFESGRYFGSKGLQVQKTRYVPLLPKVKTDPTLKQKQVVVSLVDL
>PpSTS29
MSAVTQVVETAIGCVIPSCIEFGSSMRIATSLGSPSVTQSPCVNRDVEDIARTARESIRFFLAE
LSIECVPYTQDPALEAQVASATRCWPDRERLAPHIRTGIVIAATAYAHNSLATRTLIALYTAI
GVALDEPDILESANAIGFHHSLCTETSERPSAILDEWRRILARMWDHFPRFGASCILTSTLQ
FLNMTMLENETKGKVLNRTAMPFVEYRRMTDGFPEVYTAFIWEKGRFPDVQVYMQAIPN
AMRFINFGNDILSFYKEEAAGETGTYIHDRARLTGLSSVETLREVVEETVSAWRQVCEILG
EGIARDAWNSFVRGYVTFHVHNPRYRLSELL
>Fompi1
MSRRYQTSRFSPSAKGKKTSLYTSPGDLRALFHRFSLYAGVQLESFTSPKMMQIEVPVMLN
LKESSMPMNSTVLEDCTAAVAKDAIVQFLRRLGAVLRPSFGNNRDLEERVKEITKTWPFEH
RIHPHITTGVVMANTTIAYLSDLDARAAVAAYTALITALDDPDIFHASGAQNFAQMLCDGS
ALRDDGVLGQMARVLADMGNHFPPFGTSAIIAATLRWCNGELISNPANPFCLRPLSKAFA
DYQRGLTGVPEAYAAFGWCKADFPVETDYIHVFPDICFFLNHTNDILSFYKETLDGESDSY
IHARARLTGKSVTDTLYEVMDEVITTTERIRKHFGEGRMRNAWDRYEAGYVWFHTGNPR
YRLHELVDTEYMPMY
>PcSTS01
MQTQQTSSVVSSSPRKIIIPDLVSHCTFPLRNNRHRKQATVECKRWLFKGGNLSQKKRDAF
HGLKAGLLTSMCYPNAAFPQLRVCCDFMNWLFHMDNISDDMTDRGTANTGVDVMNAL
WLLDDYTPTTRVGKMARDFWRRAAVTAAPGAQQRFRETMDFFFQSVTQQALDREAGVV
PDLESYIALRRDTSGCKPCWALIEYAYNLHLPDEVMEHPTILALGEAANDLVTWSNDIFSY
NVEQSKGDTHNMIVVVMQQDGLDLQSAVDFVGDLCQQSIDRFVYERDNLPSWGPEIDRQ
VDIYVDGLRDWIVGSLHWSFESERYFAKSGLEVKKTRTVNLLPLRTAVAEEPTQI
>PcSTS02
MQSFVLPDLFAVCPLVGSTNPHYAKAARESSAWIDGYRLFTDRKRAFFIQGSNELLVSHTY
PYADYEQFRTCCDFVNLLFVVDEVSDDQDGKGARSTGEVFLNVMCDPAWDDGSALAQM
TREFRARFMQYAGPNCARRFLVHCKDYVEAVAREAELRETGEILDVEAFKHLRRENSAIR
LCFGLFEFALGVDLPDAVFEDETFTKLYWAAADMVCWANDVYSYKMEQARGIDGNNIVT
VLMHERGVDLQTAADLVGEHFARLMDTFLQTKRALPSWGLTLDTAVAAYVAAMEHWVI
GNLEWSFETQRYFGPTHAEIKRTRVVELRPTEDAFDGDD
>PcSTS03
MSTYPALRSYRLPDLHPLCPFKARFNPHHEEAAAASKAWVLSFNALKGKKLEFFKEGGSE
LLCAWAYPYASLEGLRTACDFVNLLFTIDEISDEQNGNDALATGMSVWNTMKDDNYDDG
TVLCRMTKDFKKRFFPYAGPATTRRFLKHTEDYVLGFAREAELREKNVVLSLAAYDPLRR
ENSAVRYCFGLFGYLLGMDLPDEIFEHPLYMEMHLAAVDMVCWANDVYSYDMEQSMGH
LTNNILTVLQREKNIDLQAASDYVGVHFKELADKFEANKALLPSFGKELDDVVAHHIMA
MEAWVAGNLEWSFGTRRYFGKNHMKVRETLVVELSPPRVLDD
>PcSTS04
MPAIAPPSAPATYRLPDLHAVCGFKARFNVHYEEATAGSKAWYFSYKTLTGKRLEFFKEGG
SELLCAWAYPYAGLGQLRAACDFVNLLFTVDEISDAQNGVDALATGMSFYNTLKDDNYD
DGTVLCRMTKDFKKRFFPYAGAATARRFLEHTKNYVLGFAREAELREKNVVLDLASYEP
ARRENSAVRYAFGLFGYILGMDLPDEIFEHPIYMEMHLAAVDMVCWSNDVYSYDMEQSL
GLAANNILTVLQREKNVDLQTAADLVGSHFKVLVDKFEAAKARLPSFGKELDEIVAHHIL
AMESWVAGNLEWSFGTRRYFGKSHLKVRETLVVELSPPKYLGDL

>PcSTS06
MAPVIGTIEMPPASDGFGNPAKISPTHERSEEESLPYLCRYVITEFLRRAGIQMPTFNAASFG
DEVDRLTFAEVAKWDIGNANPRRLHHHVVSAINIAKTAYAHTPVPTQVHIAMWTALCIFV
DDFEIETAAVEQFAERFHAGGPQLHPLLDVFAGTLRAMPRFFHAHGAAGIVANTVQYVTS
TLFDKVTEEKGLEVHASARDYPLYKRARNGIGEGYGFCIFDKENFPDVSTHIQVIPEAITYL
IYVNDLLSFYKEELDGETKNFIHDRARVTGKDIEAALMDSMEDVIDAVNRGRQILQGEKE
RKAWDSFLVGYVAFHFISPRYKLERLLSGMN
>PcSTS08
MLVDLAGQLQARLRALVGVEYFKDFVAVNDERAGTEETCRNALRCFVGRVKIPARVMQP
DPTGSVTQSLRATVKAWGCIDTTSAACQKRVAAAVAITTLVFRHTRLDTQAYIAGYAFLAIS
LDDEAIGADALAAFAPRMLSGEPQGHWVLDRFIEHVQSAPTFFGAFSVAAIAACTVQFVN
STLLDRTTSNRVCLTAGSLPYVLYKRAINGVGDSFALFAWEKDRFPDDSLFLQVLPDLCRF
MEYTNDILSFYKEELAGEKDNFVHDIMTVSELPAPGVLEGLVDAAAETVQRARLVLQDSE
ERKVFESMLEGYVAFHYTSPRYRLGDLSGASPADSD
>PcSTS11
MCATLTDFLWQAEAAKHAVDSLLLRSAPLPEWKTMTFDADGTLERRIREVVADWEPAVT
SLQGFERSILISAALGMSFNSHSPMEVRLQVALYSLVIFLTDDCEIPAAALDEFMHRFYSGR
PQLHPVLDHMVEILHGMDEFFPPLAVKGIVQGTVDYMNVNAFEPHAEKLPLHPAALSYV
TARRMKHGLAEPFLMFLFDKFNYPDVSTWVQAVPDMMIYTNWANDIYSFHKEILADDVH
TYILERAEVTGKDLTETLSEIVDEAVVACDNVRKILKGTKALEAWESFVAGYAAFHRYTPR
YRLKELYGDEERDRM
>A8411
MSSTKIGKVAPFPPLPGQPWPPARNHPRWKELYRLHDEWLMKHWPFSSEKKRARIPFMNL
AGFSTWCAPASDFDRMVWGGRIAGIFFLADDYIDSGKMLDRIPGFKKAATGEGPLHPEDR
AEICHDIVFRAIKETSHPRTFRQLTQCTHEWWDSNIHEPFRNLDQYLATRRVNIAMYFANA
YFRYCLDINLTDEQVNHPLMREAEGIVSDHVGLTNDLFSYAKEYLTQSDDTNVLRMLQDF
EGLTYEQAKDTVTKKIRQKEQDFIPAGLAVLNDPELGKDPEVHRWIANLPYCMGGNNAW
SQESGRYNIGDVPGAPPFPSLSFEAEATPEDEVVDDTEESALRDAVFNVEVIPEPDFTLDID
AIESKGVRTKTQASIPPSLQLMNIPQSRVQHVGVRAIASSQRTGSDDNSLILKAVASLVRSY
EIDVQTVGKVMIYLQNSNIPVHAADLVHLLSQSEIEVNISNGDSSALLLDAITHVESNTWD
ARNVITVAVTKQSVVTMLVGPNAPIVMEPVRGVFTDNHTQKLFPSSYFTALQKSYESYLK
RLRVSLSKEIDLNEATGAASVFDYMLTDHPEKLAELFRVSPCLSKDDNALPMHPRDFQKK
VVFNGPDNGSIYDGLVCLVDAVPSYEFFEKRIAIFSESNNGTSTFFCLRAVSDTKYMRDTLK
AVGGVPRRT
>GsSTS43
MSVEQLRPFPTHFRLKDLAAISGRVFEFKLNPHEREAAEATHAWFDSRNVYHGLKKKRFL
SHRFDSYAGMSFPDADVSHLETCIAFFLWAFSFDDLSDEGALQSKPEAHQVGVDISMEVL
RNPDAPPPNFPYAAMLHDIWRRFRATASPGACNRFFHAVESWMNSQVEQARNRATDEIPS
VEEFIILRRRTIGGPIVEAMVEYSLDLHIPEQVWDHPVLQEMSKAVIDIMTWPNDLCSFNKE
QADGDFQNLVFCIMIERDCDLQTAVDVLTEMLSQRVIDYERHRAQLPSFGPEVDAELARY
NKAIEQYTQGTVVWYYHSPRYFRGQEVTGIPEIVVPVYERTTPAPEEPTPSASKSARSAPA
NVSPSLSKQAGLFVNTPVQHNARLSLLLVSLVACLLCASFVSFPSFPLNPRLVLS
>GsSTS45a
MSIEQLHPLPSHFRLKDLAAITGRVFELRLNPQEREAATAAHAWFDSHHVYQDLNKHRFIS

HAFDSYAGMAFPDADVSHLETCITFFLWAFSFDDLSDEGTFQSNPQAHQAAVDISMKVLR
NPAAPPPDFPFAAMLHDIWSRFRATASSGACNRPVHFYRLFLLLILNHISRFFRAVEGWMR
SQVEQIRNRAIDEIPSVNDFIILRRQTIGGPIVESMIEYSLDLRIPEDVWDHPILQDMSNALID
LMTWPNDLCSFNKEQADGDFQNLVFCIMIEQDCPLQTAVDILTDMLSQRLVDYEELKAQL
PSFGPEVDAELTRYIKAIEHYTQGTVVWYYSSPRYFRGQAVSGIPEIVVPVYEKSASMQDT
TLTPTAKASKPAPVVKHSPWVKADSSFVHTHVVLQCMIYLLLVLVAFVVVVLSVNKITFMI
>GsSTS45b
MSIEQLHPLPSHFRLKDLAAITGRVFELRLNPQEREAATAAHAWFDSHHVYQDLNKHRFIS
HAFDSYAGMAFPDADVSHLETCITFFLWAFSFDDLSDEGTFQSNPQAHQAAVDISMKVLR
NPAAPPPDFPFAAMLHDIWSRFRATASSGACNRFFRAVEGWMRSQVEQIRNRAIDEIPSVN
DFIILRRQTIGGPIVESMIEYSLDLRIPEDVWDHPILQDMSNALIDLMTWPNDLCSFNKEQA
DGDFQNLVFCIMIEQDCPLQTAVDILTDMLSQRLVDYEELKAQLPSFGPEVDAELTRYIKAI
EHYTQGTVVWYYSSPRYFRGQAVSGIPEIVVPVYEKSASMQDTTLTPTAKASKPAPVVKH
SPWVKADSSFVHTHVVLQCMIYLLLVLVAFVVVVLSVNKITFMI
>GsSTS26
MSDNSENILYIPDTMSAWPWPRKLNPLYEVVEAESIAWMESFKPYTPESQTAHNKGDVGR
LAALVLGDAPRESLRIAADLMHLLYIADEYTDMESAAGVQEISSTILDALHNPDKPRPEGE
SVIGEMTKEWWTRVRAVATPQAIKHFLGAMEDYLDAVGEQADDREKLAIRDVDSYLEAR
RYDSAVVACFMPGELYLSIPDEAFYHPVVKQLQDASTELVVLDNDVASYNREQATGHGK
WNILSVVMHQFGLDLHGATEWVAQHHKEVEARFFDALARLPSFGPMVDAELQEYVACV
AAWPRGNDCWRFESERYFGKKGAEVQKTRRVPLLAKRSMNPEMRRERVEVQLIEELEQV
>GsSTS27
MSDNSENILYIPDTMSAWPWPRKLNPLYEEVEAESIAWMESFKPYTPESQTAHNKGDVGR
LAALVLGDAPREPLRIAADLMHLLYIADEYTDAESAAGVQEISSIILDALHNPDTPRPEGES
VIGEMTKEWWARVRAVATPQAIKHFLTAMEDYLGAVGEQAEDRDKLAIRDVDSYLAARR
YDSAVVACFMPGELYLSIPDEAFYHPAVKQLQDASTELVVLDNDVASYNREQATGHGKW
NILSVAMHQFGLDLHGATEWVAQHHKEVEARFFDALARLPSFGPAVGAELQEYVACVAA
WPRGNDCWRFESERYFGKKGGEVQKTRRVPLLAKRPMNPEMRRERVEVQLIEELEQV
>GS02363
MAVTPASANTQDSTKEIILNFPDFISPIPYPLRCHPQEREVSRESEEWLLSMANFSEQQRAK
FLTLNAGLLSGWCYIDCTFDELRVCTDFMNFLFTLDDWTDEFDTTGTRGLAECVMNTLYF
PETYSSDTAAYRLTKSFWERMRATAGPGCQQRLMSTLDTYFQAIMQQASDRGSRNIPDLE
EYILLRRDTSGCKTGFAFIEYAANIDLPDEVIEHPIIKAMSDSTNDLVSWANDVLSYNAEQS
RGDTHNLVCVLMNQNGVDRQEAIEQAGALWEKTLNWYFECRKAVPSWGAEVDRAVALY
IQGLDDWIIANAEWSFETERYFGKEGHTVKKTRQVALLPQRPRA
>GS14272
MAIAASKPKPTPDHFVLPDLVSHCNFPLAYHPHGDDIAAESVRWLDEGCPELSPRGRKAL
YGLQAGELTAYCYPYCSTERLRVVSDFMNYLFHLDNISDGMMRKGTEELADSVMNALWF
PDRYMPTACDGKEQPADEVSAGKLARDYWTRCTRDAKPGPQARFKENLELFFEAVYQQA
RDRDTKLIPDLDSYISVRRDTSGCKPVFDLIEYAMDIDLPEHVVRHPVIQALNQGANDLVT
WSNDIFSYNVEQARGDTHNMIVILMELRGFDLQTSVDFVGELCRQTIDTFMENQQNVPSF
GPRLDRDVALYIQGLQDWIVGSLHWSFMTERYFGKSGAEVKKHRIVRLLPRRPKRIPVSA
>GS11330
MLSTLPPPASGVHHPLITTTKETEDPLSEVKVLISDFLDRSNYKSPQSPCDLELRKKITEELS

TWPSDINPTAISKIIDGCCVFVETAYGHTTHEHRCFIAFYIAFCIYIDDLGERDLDAIKLFTSR
LVKGETQPDPILRRLAEHLGCVYDLWTPFGADAIIAGTLDAITATYLEFTTQEMVVKPAST
RFPYYLRTRAGLGPPAIHFLFMKDWRATPESYLQIIPDMEHWTLGTNDILSFYKEELAGER
NNYVHLRASAEQSSAANVLRQLVEEVLESAAKIDALTSEDPELAALWKRYQQGYIEESLK
AQRYRLAELGYQA
>GL26009
MAVTPASANASDSTKEIILKFPDFITPIPYPLRCHVQERQVSRESEEWLLSMANFSEQQRAK
FLTLNAGLLSGWCYIDCTFDELRVCTDFMNFLFTLDDWTDEFDTTGTRGLAECVMNTLYF
PDTYQSDTAAYRLTKSFWDRMRATAGPGCQQRLMSTLDTYFQAIMQQASDRGSRNIPDLE
EYILLRRDTSGCKTGFTFIEYAANIDLPDEVVEHPIIKAMSDSTNDLVSWANDVLSYNAEQS
RGDTHNLVCVLMHQNNVDRQEAIEQAGALWEKTLNWYFECRKAVPSWGPEVDRAVAM
YIQGLDDWIIANAEWSFETERYFGKEGHTVKKTRQIALLPQRAHA
>GLSTS6
MSVEQLRPFPTHFRLKDLAAISGRVFEFKLNPHEKEAAKATYAWFDGRNVYHGLKKKRFL
SHRFDSYAGMSFPDADVSHLETCIAFFLWAFSFDDLSDEGALQSKPEAHQVGVDISMEVL
RNPTAPPPNFPYAAMLHDIWRRFRETASPGACNRFFRAVESWMNSQVEQARNRATDEIPS
VEEFIILRRRTIGGPIVEAMVEYSLDLQIPEHVWDHPILQEMSKAVIDIMTWPNDLCSFNKE
QADGDFQNLVFCIMIERDCDLQTAVDVLTEMLSQRVVDYERYRAQLPSFGPEVDAELARY
NKAMEQYTQGTVVWYYHSPRYFRGQEVTGIPEIVVPVYERTTPAPEEPTAPASKSARSAPA
NLAPSSSKEVGLLVNAPTQHNARLSLLLISLVACLLCASFISFPSFPLNPRLVLSI
>STC4
MVQFRIPDLLSCLPACIKATNADNDILQAGLVVIDQCHLTDHYKKDLKRAQIPHLAIRAFP
GSDLKYLRICVEYLIAAFLLDRLTDKPATAAQAQEWADIYKQYFRKTLQGTKGPARINQYL
TKKCDIYPQAFSKTFEKTKGPAEIKYLTSHMSNTIKDPYWSCLVENNILLADGMAKEAVDR
ENPGTEMDLETYIKVRRDTIGARQLFDLGRWIHELNITPETLTHPDIVRMEEQFIDLISLAN
DLYSYKKEYLAKDAKHNYLTIALRDPTADLHENDLQGAINYTYDKFCQVLTDLEHQKKV
LPRFGKSEEAKVDKYFWLMMNVVIGTIQWSLECERYGHFVDVDGPNQGDVVFNL
>STC9
MFRFDHPSSFILQNICDITGAVFELKENPLREQANTAVLKWFKGFNVYDKAQGEKFINAGR
FDIFAALSFPEADIEHLTTCLAFFLWAFSTDDLSDEGEYQSKPEKVRRGHEISCSILHDDSAP
QPSYPYAGMLWDLLRRLRANGKSGMYKRFKQAFLDWSSSQVQQSLNRNLDRIPPVDEFI
LMRRCTIGAALVEAMVEYSLDLDIPSFVWEHPVIIGMSQVTSDIMTWPNDLCSFNKEQAD
GDNQNFVCVLQHAHNLNLQEAVDLLTKMIADRVQDYVKLKKRLPSFGPDIDPAVHKYVD
ALEQFVQGCVVWYYSSPRYFPDIDPRGKSKAEIHLLSKPISSDPVQMH
>STC15
MSAATSQLLPSALATKIILPDLVAHCDFTLRYNRHRKQITRETKRWLFKGGNLNGKKRDAF
HGLKAGLLTAMTYPDAAYPQLRVCNDFLTYLFHLDNLSDDMDNRGTRSTADVVLNSLYH
PHTYYGPERVGKMTRDYYKRMIVTASPGAQQRFIETFDFFFQSVTQQAIDRANGVIPDLES
YIALRRDTSGCKPCWALIEYANNLNIPDEVMEHPIIVSLGEAANDLVTWSNDIFSYNVEQS
KGDTHNMIPVVMNEEGLDLQSAIDFVGNMCRQSIDRFVEDRTNLPSWGPEIDKDVAVYV
NGLADWIVGSLHWSFESERYFGKTGREVKANRVVNLLPRRA
>AbSTS05
MPRPQQFILPDLLSSCPLEDGLNPHYREAAAESRAWINSFNIFSNRKRADFIQGLNELLCSH
VYCYAGYEEFRTTCDFVNVLFVVDEISDEQSGKDARATGLSYAESMRNADWDDNSVVAK

ITKEFRARLIRRAGHNNFRRFVASSDAYTRCVGREAELREAGEVLSLEEYMPLRRNNSAVL
LCFDLVEYILGVDLPESIYQNATFLKAYWAACDHVCWCNDVYSYNVEQSKGHTGNNVVT
VLMNDRQIGLQEACDYIGDRCRQFMNDYLAARDELRATVGGDASRFIDALGYWIIGNME
WSFESPRYFGHEHDEIKRTLTLTLKPSEVPEEVDSDSDCE
>AbSTS07
MRAIEQLSNQPLSHKALEDGVRDVISDFLLKTNQTPKAPEQPDALVFERECWAEADRRGY
DMHYLSKHLPVGILVSNAAYFYQSFELRLYIAYYTGLLLCVDDNFDIQSDGIARFMERYQR
GECHPTDVLNNLADLLAETSTFFDPVATNLIMCATFSFMNAMVIENITAGTKIPSQAKRYP
DYLRRFSGISKAYATFIFRPSVSAAQYIHALPELEDIINYVNDITSFYKEELAGEDENTVSLL
AKLNNRSKLDQLRVLSDSVAESHRRTLAILKGRDDAEGDYLLFWSGYIPFHVSAKRYRLM
ELGIH
>AbSTS09
MGAVGQLSNQPLSREALEDGVRDVISDFLLKTNQTPKAPEQPDAPVFERECWAEADRRG
YDMHYLSKHLPVGILISNATFFYHSFELKLYVAYYSGILLCVDDNYDTQSRVDGIARFMER
YQRGERHPTDILNNLADLLAETATFFDPVATNLIMCATFSFMNAMVIENITAGMKIPSQAK
RYPDCLRQFTGISKAYAAFIFRPSVSAAQYIHALPELEDIINYVNDIASFYKEELAGEDENAV
SLLARLNDRSKLDQLRVLSDSVAESHRRILAILKGRDDAERDYLLFWSGYIPFHLSTTRYR
LMELGFN
>AvSTS01
MQSPTQASYRLPETLAGWPWPRMINPHYEEVKAESRAWFHSFRAFGPKSQDAFDKCDFW
LLASLSYPFTDKARLRTGCDLMMLFFVFDEYTDLSHGNDVRVYADMVMDALRNPHKPRP
AGEILLGEVARQFWELAVKTTTPTAQKRFVDAFTRYTDAVVAEARDRDETHVRSIDEYFNI
RRYTIGAEPSYVPMELAMDIPDEVFFHPTVVKLTQLVTDVILLDNDLCSYNKEQANGEEL
HNILTIVMAELKVDLNGALDWLERRHAELNEAIIETWNSLPVWDEDIRDDVDEYLLGVV
GWVRSNDSWNFESQRYFGTDGLEIQKHRMVTMRPRKAGLGCPVEGDPLQELMV
>AvSTS03
MPEKFYIPNCLENWKWPRALNPYYEEVKAESAAWARSFGAFSPKAQHAYDRCDFNKLA
CLAYPLLDKAGARIGCDLMNMFFVYDEYSDVAPADEVQVMADIIMDALRNPHKPRPEGE
WVGGEVTRQFWELAIKTASPQSQKRFIKTFGTYTQSVVQQAADRDHHYVRTVQEYLEVR
RDTIGAKPSFAILEVGMDIPDEAIEHPIIQELTILSIDMILLGNDIASYNLEQARGDDNHNIVT
IVMHQEKTDIKGAMDWVVQYHKTLEDRFMELYAQVPSLDFGERVNKELAVYVDGLGNW
VRASDQWGFESERYFGKKAPEIQKTRWVTLMPKERTDEIGPQIVDGSML
>AvSTS06
MSTQIQFRLPDPVEICHWPLPRMLNAHYAEVKAECVAWIHSFNALSPKAQKAFDKCDFSL
LASLIYPSLDRQHLRTGCDLMMLFFVFDEFTDKEDGKGVRKYVDIVVDAIEHPDRPRPAG
EHVLGEITRQFWERAIQTASASSQRHFVKTFKEYAEAVIEEASDRVSDRVRSIDDYLALRRL
TAGPYPGFFPCEIRVDLPDDVFYHHSVANLTRLVAESVVVTNDTYSYNIEQAAGHQGHNIV
TVVMREKHLSLHQALEWVGNYHAGILTEFLESRKKLPSFGEELDAQVADYVEGLAHGVR
GLDNWCFESGRYFGSKGLEVQKHRTVGLLPKVVQHDVATPMMALPVSDLDKMEVGESD
LEKHQSSGWRYFDCFSCLSGRWMYL
>AvSTS07
MQSQIPLTHFRLPDPAANWPWPRTLHVNYEEIKAEADAWLHSFNALSSKAQRAFDKCDFS
LLGCLLYPHLDKERARTGCELMILFFIFDEFTDQEDGPGVRRYVDIVLDALRNPHVPRPVG
EHVLGEITRSFWERAIKTATAASQRHFIQTFSEYAEAVILEAADRASERVRGIEDYLALRRLT

AGPYPGFLPCELRIDLPEAVYNHPALANMRRLVAESIVLTNDTYSYNIEQAAGHDGHNIVT
VAMRELHLALPAALEWVGAYHANILAEFVECRGELPSFAAEDAQIVEYVEGLAMGVRGL
DAWCFESARYFGTRGREIQRERVVGLLPKTVIVGGLATPMMAGPAVEGNSVVV
>AvSTS09
MLTSPGHFYLPDLLALSTPFHSSTNPHYKEAAAESRAWINSYNVFTDRKRAFFVQGCNEL
LVSHTYPHAGYAQFRTICDFVNLLFVVDEVSDDQSGADARKTGEVYLNAMRDPEWDDGS
PLAKMTKEFRARLLGSCGPRSFARFLRHSEDYINCVALEAEYRERGEVLDMEAFKHLRRE
NSAIRLCFGLFEFSLGIDLPDEVFQETTFSSIYWAAADMVCWANDVYSYNMEQAKGHTGN
NIVTVLMKAKGFDLQAASDYIGIYYAELMNRYIADKARLPSFGPSIDTDVQRYVTAMENW
PIGNLVWSFETNRYFGPRHAEIKRTRLVILKPREEDT
>LnSTS01
MSPSSEIYFTIPNTLQNWPWPRHINPNYNVCKAESSAWCEGFKAFTPKAQKAFNKCDFNL
LASLAYPLLNKEGCRVGCDLMNLFFVIDEYSDVADQCGAREQADIVMDALRHPLKPRPEG
EWVGGEIARQFWENAIRTATPSAQRRFIRMFDSYLNSVVQQAEDRTHNYIRDIQSYFDVRR
DTIGAKPSFAINEIHLNISDEVMEDPIIKTLTSTSIDMLIIGNDLCSYNVEQARGDDGHNLVTI
VMNERKIGLHAALQWISDLHDRLAAEFLEAYKKLPSPIDPDVATYVDGLGNWVRGNDV
WSFESERYFGVRGLEIQNRRIVQLLPKEKGEQGNTGLIPSTQGISVSTIKPSGFIQNILSSIWI
KWGVTFLVLMLSVASLRSIIILHRYELF
>LnSTS02
MSPSSEIYFTIPNTLQNWPWPRHINPNYNVCKAESSAWCEGFKAFTPKAQKAFNKCDFNL
LASLAYPLLNKEGCRVGCDLMNLFFVIDEYSDVADQCGAREQADIVMDALRHPLKPRPEG
EWVGGEIARQFWENAIRTATPSAQRRFIRMFDSYLNSVVQQAEDRTHNYIRDIQSYFDVRR
DTIGAKPSFAINEIHLNISDEVMEDPIIKTLTSTSIDMLIIGNDLCSYNVEQARGDDGHNLVTI
VMNERKIGLHAALQWISDLHDRLAAEFLEAYKKLPSPIDPDVATYVDGLGNWVRGNDV
WSFESERYFGVRGLEIQNRRIVQLLPKEKGEQGNTGLIPSTQGISVSTIKPSGFIQNILSSIWI
KWGVTFLVLMLSVASLRSIIILHRYELF
>LnSTS04
MALITSLKSTERTTFVLPDLLANWPFNPEPNPRQDIVAQSAAWVESFNPFDAKAQNAFNR
CKFGIFASLAYPHATGAHFRVACDLMNLFFVFDEYSDRADGEAVGKQAADIMNALRYPDT
IPPEGDSLLGAMTRDFWLRTMKCASESSAQRFIRNFDEYTDAVRQEAADRDAGCIRSVPE
YFKIRRGTIGVHPSFDYYLLRDDLPDECVNHPDVQRLASAAVDMTILANDVYSYNKEQA
KGEDSHNLVAVVMKEHDLTVQEAMDYIGDLYNHIRKQYCEKFQDLPRFNDDVDGLVREF
CYGTGIWVTTNIKWSFASERYFGKEGMEIMKHRTVTLLPKVDKLFK
>LnSTS09
MNHTTTQILLPDLISMLPLEGATNPHYEKGAAESRAWINSYNVFTDRKRAFFVLGSNELLV
SHAYPYAGYDVFKICCDFVNVLFVFDELSDEQDGKDALTLGNIFVNAMTDPLWNDQSKFS
RMTKEFRSRYTKLAGPNTTARFLKYWESYCAAVITEAELREKGQILDVDSFMELRRDNSA
VRLCFGLIEFCFGTDLPDEVFEDPTFLKIYWAAVDLVCWANDVYSYDMEQSKGISGNNIVT
VLMHDKNMDLQTAVDYVGTYSKELVDRFMDLQAHLPSWGSAVDSQVALFIKGLGYWVK
GNLDWSFETQRYFGPKHMEIKNTLLVTLRPLECPEETDSESDTE
>LnSTS19
MALYSLRSLLPTLFSFAISPISPLDSSMNYHTYRLSDEEIERSIYRDFLRKMEYCKPDLHDAE
KTLEQALRSEMYSRNMQCPQLEKTVHLAARLVELAYPDCTFEEQKVIALVNWFVIYLDD
VDSEPCAAFVPRLLRGQKQLDPVLDAFADVLNSMHDYYDSLAANQIVTSILNFINLTPVES

QIAAGTFTVPSRSRRFPGFLRDRSGLGVPFALFAFPKSQGLDTAAYLRALPDMDFWICAAN
DLLSYHKEMLAGETNNYVSSRALVEKKTPLRVLVELERELEESRNVIHATLSSHPAAIKAW
RAFEVGGVAWHLEQKRYKLKDLALDQQVTARG
>LnSTS20
MAPVSLIRPTSANVVQDLNKTGILRRFLQGMAYCPSNISGTNEELEKIMRAEMNNRNIRCP
QLEKTLHLAASLIELGYHDCTLTEKTNIALYNWYLIYIDDMSSKDTGPFMAFQERFLRRMP
QLNPVLEALVGVLMRIYELYDMPTANSILSATFNFVNSTCIEPEIETLPLTRGLVRFPWFLRD
QTGVAIAFALLLFPKSKNVGVTDYIQALSDMNFWISVTNDILSFHKEELAGERANYVYNR
AYIEDKVPLSVLAEMSQELFESRNAVYAALEHCPQAADVWHTWEQGYVRWHIDQKRYK
LSELSL
>LnSTS25
MSQDTSKAKFEMAAVLKEFLQETAYHPPTIPSTNEELENKMYEYMHGRNLACPQLERTLH
LAASLIEIAFPECTLSEKTVIAIFNWFIIYIDDTSPGDVSPFVAFGPRFFSQTQQLDPVLDAFV
DILQRICDQYEPATANYILASAFNYISSTCIEPEIEAHAVVPGVIRFPWFIRGQTGISLAFALM
LFPKSKGVSAVQYIQVLEEMNFWISGVNDLMSFPKEQLAGERNNYVHVRARTEEKSPME
VLAELNQELHMSRKLIYAALSLIPGASTIWSAFENGYIKWHLIQERYKLAEMDLTG
>LnSTS27
MSTVTQLLNNFVHVVSRAGSLITPDFLNGEFYDAPLSDAGDPPAKDDVTDLMVTVGQILG
KMLHDCHIPYCAVSFDYGLMKAPVAEAERRGYALEGPNSMRQALFLGVYLTSTMYAHVT
DDDALRTYIIFYIMFMFYVDDKYFEQSDKESGLLHFIARFNQNQPQAEPSLTNFADFLRDD
TAAVFEPIGGGIIITSTLNFINGMILETSVKLGQISPYAQNFPYFVRNKSGFPEACVILAFPRD
MPVRQYAQAFPEICDYLCYVNDIMSFYKEELVGETENLVSLLAAVTDSKVEHLLSTDSPAP
VTRNDKYGVLRRLSAAVASGHKKTLQILSGNPVAQDIYLKFAIQYVTMYISMRERYKLDE
LRFGDKA
>PoSTS01
MSAIASSSAQAAPPSKIVIPDLVSHCTFELRVNRHRKIASAQSKRWLFRGDNLTGKKRDAY
HGLKAGLLTSMCYPNAGAPQLRVCCDFMNYLFHLDNLSDDMDNRGTKSTADVVLNALY
NPKMRQTKRVGKMTKDYWQRLIRTAAPGAQQRFIETFDFFFQAVSQQARDRAEGSIPDLE
SYIALRRDTSGCKPCWALIEYANNLDIPDEVMDHPIIRSLGEAANDLVTWSNDIFSYNVEQ
SKGDTHNMIPVVMHEEGLELQAAVDFVGALCKQSIDRFVECRANLPCWGPEIDRQVAVY
VEGLADWIVGSLHWSFESERYFGKSGLEVKKNRVINLLPRRS
>PoSTS02
MSTLISPDHFILPDLVSDCTYPLRVNDNCEEVARVSEQWLLNAANHNERKRRAFLGLKAG
ELTAACYPDADAFHLRVCVDFMNYLFNLDDWLDEFDVEGTRGMHDCCIGVMRDPLNFE
TDKRAGIMTKSFFSRFIETGGPGCTERFIHTMDLFFKAVAIQAADREKDVIPDFESYITIRRD
TSGCKPCFALIEYASRIDLPDEVAEHPLIRSMEEATNDLVTWSNDLFSYNVEQSRGDTHNMI
PVIMHQRDLNLQEAVEYVGALCKSSIQRFETDRKNLPTWGPEIDRDVAVYVEGLQNWIVG
SLHWSFDSERYFGTSGLEVKQQRIVKLLPKVPS
>PoSTS03
MVPVASFIQEPSRAPSSFILPDLVSHCKFPLTYHPQGDAVALESVTWLDRLCPDLSPKARKA
LWGLQAGELTAYCYPSCSPDRLRVVSDFMNYLFHLDNISDGMMTRETDVLADSVMNAL
WHPEEYRPTRSPGKEQPAEELDAGKIARDYWSRCIPGAGLGVQARFKESLQLFFEAVNVQ
ARARDAGEVPDLESYIDVRRDTSGCKPVFDLIEYAMDFELPEEVVNHPVIKALNQGTNDL
VTWSNDIFSYNVEQARGDTHNMIVILMKYHGHTLQSAVDYVGELCCQTINNFCANRQVIP

SWGPETDRMVQEYVQGLQDWITGSLHWSFKTHRYFGTNGAEVKKTRLVKLLPLKNGAN
PCAGEATK
>PoSTS05
MSSQPTQIVLPDLLAMCPLKGYTNPHYKEAAAESVAWIDSYNVFTDRKRAFFVQGCNELL
VSHTFPYAPYEQFRTCCDLVNLLFVIDEVSDDQSGKDARATGQVFLNALGDPEWNDGSLL
SRITKDFRARYFRLAGPNSSRRFLKHCEDYINAVSTEAELRERGEVLDIEPFTALRRENSAIR
LCFGLFEYALGIDLPDEVFQDKTFQDMYFAAVDMVSWSNDVYSYNMEQAKGHSGNNIVT
VLMKSKGMGIQEAVDHVGVHFQQIMDIYMDSKTRLPSWGSEVDVNIARYVEALGHWAK
GNLDWSFETQRYFGAEHLEVMATRVVTLRPHGSPEDFDE
>PoSTS06
MTSTTKHPLPSHFILPDLLDQWPFETEPNPHQEIVDDSARWVESYKAFSPKAQDAFNRCNF
GIFASLAYPRSEGTHYRAACDLMNLFFVFDEFSDAENGDVVRQQAADIMNALRYPDKIPA
GGDSILGAMTRDYWKRTLEVSSKSSAERFIRNFDGYTDAVRQEAVDRDEGRERSIEEYME
LRRGTIGVYPSFDYFLLEDDIPDEYIDHPAVASLALGAVDMTILANDVYSWNVEQCRGEDR
HNLVAVAMREKGLSVQEAMDYVGTIYAGIRDKYVKEFNELPQFPEKYDKLVKDYCWHM
GNWVTTNIKWSHFGERYFGKKGREILKHRTVEVMRPSVLGWVMRKSYPLIVTVKYAPRE
YYYIAVTFLMLLLALLSSPSRARILGHSSFPSSF
>PoSTS11
MASHYRLPNLLALFNTDGARVNPRFHELDSQFNQWLDTLTLDAGFVETLKQIQMPVLISH
AYPEASLGQLRTCLDYLTLAFIFEEITENTSSSQSQRWADLYMGMYRGTASVLSNIVPEETD
HPLFPVMTSLAHNVMSSLEPVFHESFITENLASVQAIVQEAIDREVDDAPSRQPRTLEAYYI
NRLATVGLMPFLILAQWTGGIRLPAFIQDSHSVQTMSQAAVEMVFLANDIYSYKKEKMAG
ATQNNVITVIIEDPSTSICEGNLQGSFDYSERLFQDALSRFYTHREMLLENVSDQQNYTADI
NKFSRAMMDCVVGNIKWSMICRRYAVFECEQARNNEVVKI
>PoSTS16
MPVNIRDIVALHLRRCNIPYEVPVPDVKLQAACLNYAKNHGYEIGGKKTLGPCIPGGVVM
ASNAFGHIADLPTRVIIGIYTAFMIYLDDISSSDIEAVAQFNQRFYRAEPQLDKVLDDFAQLL
RDFPNYFCTAGSDMIVTSTLNFVTSLFMDVETEGMDVDGNAKRYPRFARILNGAALAYTL
LVFPKSLPISSYIQAVPEINVYVENTNDVLSFYKEQMAGDDINYASLLSHSYSVTKYEALIH
LSNIAVDANERILRLLEPVPEALEAYTRFRNGYVRFHTGLGGRYKLAELKLTNISDYQDRL
GAHVNQTACKVCQRNLCN
>TvSTS01
MAVIAAAPRPKRIFFPDLIAHCPYPLRYNPHCEAASAESKVWLMSGCRLSKKKRAAFHGL
KGGLLTAMVYPEADYDQFRVCCDWINYLFHLDNICDEMDDRTTVSTAGVIIGALRDPHGF
RPASAVGRLTQSFWRRMSATASPGAQRRFIETFELFFRAVAQQARDRASGNIPDLESYIAMR
RDTSGCKPCWALIEYANDLDLPDWVMDHPCVRGLEEAANDLVTWSNDIFSYNVEQSNGD
THNMIVVVQTQEQLDLQSAVDYVGDLCLGCVDRFETLRAALPSWGPQIDDQLAVYVQGL
GDWMIGNLVWSFETERYFGRSGRKVRRALAVALLPRRK
>TvSTS05
MSSPSSFVLPDLHAVTPFKGSFNPHYPEAAAESSEWVNSYKVLSDKKRAFFLQGGSELLC
AHAYPYAGYQQFRTTCDFVNLLFTVDEISDDQNGKGAYETGLTFYNAMSNPAYDDGTVL
CKMTKEFTARLLEHCGPQTYRRFIKHCKDYIEAVAVEADLRERGEVLDLEAYQTLRRENS
AVRFCFGLAGYALGIDLPDEVVEHPAFMAMHLSTVDMVCWSNDLYSYNMEQAMGHTGN
NVITVLMQHKGLDLQGAADYTGVHFKGLIDTFLDAKRSLPSWGPKLDGEVAQYAMAME

TWVIGNLNWSFETQRYFGHARHEIKRTRVVQLYPRRIVEESSDEEDN
>TvSTS06
MTSTTKHPLPSHFILPDLLDQWPFETEPNPHQEIVDDSARWVESYKAFSPKAQDAFNRCNF
GIFASLAYPRSEGTHYRAACDLMNLFFVFDEFSDAENGDVVRQQAADIMNALRYPDKIPA
GGDSILGAMTRDYWKRTLEVSSKSSAERFIRNFDGYTDAVRQEAVDRDEGRERSIEEYME
LRRGTIGVYPSFDYFLLEDDIPDEYIDHPAVASLALGAVDMTILANDVYSWNVEQCRGEDR
HNLVAVAMREKGLSVQEAMDYVGTIYAGIRDKYVKEFNELPQFPEKYDKLVKDYCWHM
GNWVTTNIKWSHFGERYFGKKGREILKHRTVEVMRPSVLGWVMRKSYPLIVTVKYAPRE
YYYIAVTFLMLLLALLSSPSRARILGHSSFPSSF
>TvSTS07
MSRSLRLPDTLSRWPYPRRINPAYEEVSAESAAWLRSFHAFSDQAQVAFDKCKFGLLASLT
YPNVDKDHLRAACDLMNVFFVFDEQTDIADTTRTRELADIVIDAVRHPDRPRPEGEPIVGE
ITRQFWAHACVNSTASGRARFEQEWTRYVESVVGQAEDRDAGRLRTTEEYLELRRFTIGA
DPSYALAMARVDLPLAVSELPVFRKLRGCITDMLIFDNDLLSYRKEYAAGDDMHNIITLV
MNEKQIDVDAAVEWLAAEHAKRVDEFFVLWPQASAMSFGSDELNQAVATYLDHLVNWP
RGDECWSFESGRYFGKDGARVKKERVIELKTRD
>TvSTS12
MSVEQLRPFPTHFRLKDLTAITSPVFEFKTNPHQEAAAQATNVWFERRNVYHGLKKQKFL
SHRFDSYAGMSFPDADAAHLETCIAFFFWAFSFDDLSDEGALQSKPDAVQVGVDISMEVL
KNPAAPAPNFPYAAMLHDIWRRFRSTASPGACNRFYRAVESWMNSQVEQARNRSTDEIPS
VEDFIILRRRTIGGPIVEAMVEYSLNLQIPEYVWDHPVLQEMSKAVIDIMTWPNDLCSFNK
EQADGDFQNLVFCIMIERDVDLQRAVDILTDMLAQRVADYVRYRDQLPSFGPEVDAELAR
YNKAMEQYTQGTVVWYYYSPRYFRGQDVSGKTEIVVPVYERAAPVEEAAPAPHVSTTDS
EKGAVYASFRFPFSAFPHLSLLLLSLFACLLYVNLPSLGLRLALP
>TvSTS14
MPYIQDACSSPLPLCASVDAITEIKDILKDFLRRLNYRSPHTPANAKLRAEVTAEIASWNAD
LSPSFKHGLAETCYTIAESAYAHTSYEHQRIIALYTTYLTYVDDLGGRDLDALGEFGRRLL
AREALGDSALDRLVTNLQDMYAYYPRLSAHSIAVSTLDFFVGSYVEATGKEMAVAPGATK
YPGYMRMKTGIGAAYALFNFVKDWRDPADHFFLQLIPEIEFYTDTINDILSFYKESLAGET
DNLIHLRAAAEQKDPLSVLRDVVEETLESIRKVEVLTAADPQLAKICRSYVVGYVEFHFRA
KRYRLEDLEMEY
>TvSTS16
MVAEVLPSPANPFLVYANGVSPILPTSLSHIDGHVYRSGSNDSNGLSRLLVTGPQGLPPAITP
EAIQEMKAIMQDFLQQCNYEDPSTPKDIELRERMALEMAAWSLDLPATFIEQVHETSCHFI
ETAYVHTTLEHRFFVARYCAYFLYADDLPGRCLDALIQFPRRFANREQQLDPILNRLAEMIR
GAHELWTDVGASAIIAGTLDALTAFYIEYTTCGLAVKPQAVRYPYYLRLKSGIDPPFVAFIF
MRGWRDTAESYVQMLPEIEYWIGATNDLLSFYKEELAQETNNYIHIRAAMEQTTGLVILR
KLADEILDTTRRIEGLAAEDAELAALWHGYKQGFLEFQIKAPRYRLSDLGLTE
>DS3
MTPTDIDNTSSHSNLYTAIIFDLGDVLFTWSLSSNPPLPEKLLRRILSSSHWFEYEKGNINEA
EVYSLVARDFLVDPAALKNTLQVAQDSLQSNKKMLGVIQELKEAGLLIYAMSNISAPYLEI
LERKATPSQWALFDHVFTSASAHQRKPNLGFFKHVIERTGIDPSCTIFVDDKLENVLTARSF
GMHGIIFDNESKVIKDLKNLCYDPVLRGKRFLTSHKKNLKTVTSNGIEFMDDYSQLVILLA
TGDDSLVDYVKSPGQFNVFPDGTLFTTEVYPNDLDTTAIGLTVTDHVDAGTKHKIMDEML

EYRDSDGIIQVYFDHSRPRIDPVVCINVLNLFCENGRGHELPETLDWVEQVLIHRAYISGTT
YYIGADVFLFFLSRLLQNSAEVRRRLGSIFKERVIERFGVKGDSLSLSARMIAATVAGVIDE
GALKNLLSMQCEDGSWDDSWFWRWGMSPIMAKNDGVTTALAIWAIERVQSLRKE
>SiTPS
MPSVSPATIRLPDILGAMDRFELRTHPDEREVTRASNEWFNSYNMMPPALFEKFVKCDFGL
MTGMSYPDTDATRLRITCDYMSILFAYDDLMDLPSSDLMHDKIASDKAAKIMMGVLTHP
HKFRPYAGLPVATAFHDFWTRFCATSTPKMQKRFTDTTYEYVMAVKNQCGNRQSSRCPTI
EEYVALRRDTSAIKVTYACIEYCLNIDVPDEAFYHPSVAALQEAGNDILSWANDVYSFDNE
QSSGDCHNLVAIVAINKNITVQAAMEYVMGMIDSAIERFFEECANVPSFGPEVDPLVQAYI
KGVELYLSGSVFWHLESERYFGARVQHVKDTLMVELRPLDEGAKPAFDLMYKLPSNLTP
EVLSAAAVSAAPAAPAPVASPAPQPEILSPTPISPINVNFPLGNVACPPPSYETQRVLAKMVA
ATVEEKQRLAYSQPAEQYYSPAPQYYPSQPVEKFQQTNVLETAFKGSNSELTNILVIASVL
MAGSPMALVPFVPLLALLLLPNETPVAPVA
>IIIS
MSQQSEAQTHAASEAPAQQPEMLYLPETMKNWPWPRAINPYYEEVTRESNAWFKSFKPF
NERSQYGYDLCDFGRLASLAYPYVSREHLRTGIDLMNVFFVVDEYTDVEPLPVVKEMIEI
VIDAIKNPSKPRPEGEIILGEIARQFWARGEKTATPEAAKHFVEAFTDYLNSVIVQASDRDN
DKIRTVDEYFQTRRENIGARPSYVPGELHLSIPDYAFYHPVMKELEYLIADLIILDNDIASY
NKEQATGDDRHNILTIVMHQFNLSLPDAMKWVVKYHDEVEAKFLDGLKRVPSWGKEVD
DQVAVYIEHXLLVGLAATTAGTSRVAGISVARDLRSRRLVWCPFSQRESWLLTFTRKMSKF
LL
>LbSTS4a
MAPTTQQQLHIPDLLSILNIQSDTNPYEDVVRPETEKWVSSYGIFTGTSWDKFLASDVPRF
SSRTFSYADRDKLRDCTDFCLLMFALDDITDDEDQKEGTLTGQACIRTMQSDGPLLSSSPG
ERMIKDYRARFFKSTGPVLQRRLLKDWEATFNAFPKELEVRESKEVLSIEEYTLIRRDNCA
LRMGFTHIEYALGIELPDEVYQNPVFNEMYLAALDMAWLLNDVYSYGKEHAKGQATWN
YLTCVMHEKSIDLQTAADYSGIKFRELYDRFIDGKSRLPSWGKPLDSDVAVFLEGLGIWLA
GTLRWCFEMPRYFGPRYEDVRFTYLVDLGVFEG
>LbSTS6
MLTDLAYWVRTFLAFDVGTIKGAPLEPLLEKLSTQSVIADPHDEIKNAVTNLLAECNLPFP
VTPYNHEFHNACCQVARSKGYAMETYPGARSLHPFMPGGVVMATTAYAHLDNKSTQIFIA
LYTAFLIYLDDVFQHDIELVYCFNERFILCQPQDDPVLDGFASLLREFPQHFGRVVSNIMTT
STLNLVTALLLEHETQNMKSSLDAHNYPTFSRVMSGASETYALFAFPPQIPMPFYIQALPED
MMFINNVNDVLSFYKEDSAGETVNRVSNLARCHGVSKMEVIHRLSQDAATCHSQVLRILS
PHRDAHTAYANFSQGYIGFHASLARYRLNELFS
