# Supplementary material for: Molecular and Functional Analyses of Characterized Sesquiterpene Synthases in Mushroom-Forming Fungi
Source: J Fungi (Basel). 2023 Oct 14;9(10):1017. doi: 10.3390/jof9101017 (PMC10608071; doi:10.3390/jof9101017)
Supplement: Supplementary file 1 [file jof-09-01017-s001.zip › jof-2583866-supplementary/Supplementary Figure.pdf]

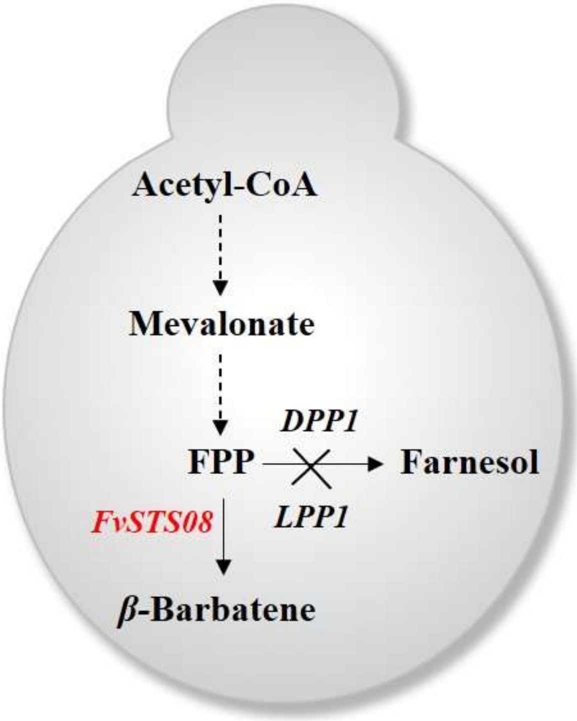

Figure S1. Scheme for the biosynthesis pathway of *β*-barbatene in *S. cerevisiae*. Endogenous genes are shown in black italic font. Heterologous genes are shown in red italic font. *FvSTS08*: *Flammulina velutipes β*-barbatene synthase; *DPP1*: phosphatidate phosphatase DPP1; *LPP1*: phosphatidate phosphatase LPP1; FPP: farnesyl diphosphate.

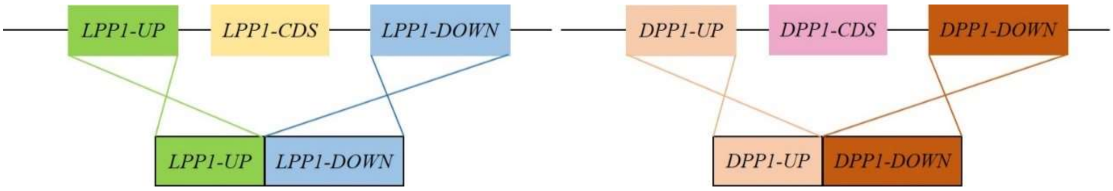

Figure S2. Preparation of DNA donor expression box of genes to be knock out.
